# Supplementary material for: The Modulatory Role of MicroRNA-873 in the Progression of KRAS-Driven Cancers
Source: Mol Ther Nucleic Acids. 2018 Dec 13;14:301–17. doi: 10.1016/j.omtn.2018.11.019 (PMC6348737; doi:10.1016/j.omtn.2018.11.019)
Supplement: Document S1. Figures S1–S11 and Table S1 [file mmc1.pdf]

OMTN, Volume *14*

Supplemental Information

The Modulatory Role of MicroRNA-873 in the Progression of KRAS-Driven Cancers

Hamada A. Mokhlis, Recep Bayraktar, Nashwa N. Kabil, Ayse Caner, Nermin Kahraman, Cristian Rodriguez-Aguayo, Erika P. Zambalde, Jianting Sheng, Kübra Karagoz, Pinar Kanlikilicer, Abdel Aziz H. Abdel Aziz, Tamer M. Abdelghany, Ahmed A. Ashour, Stephen Wong, Michael L. Gatza, George A. Calin, Gabriel Lopez-Berestein, and Bulent Ozpolat

Supplementary Data

Supplementary Fig. 1

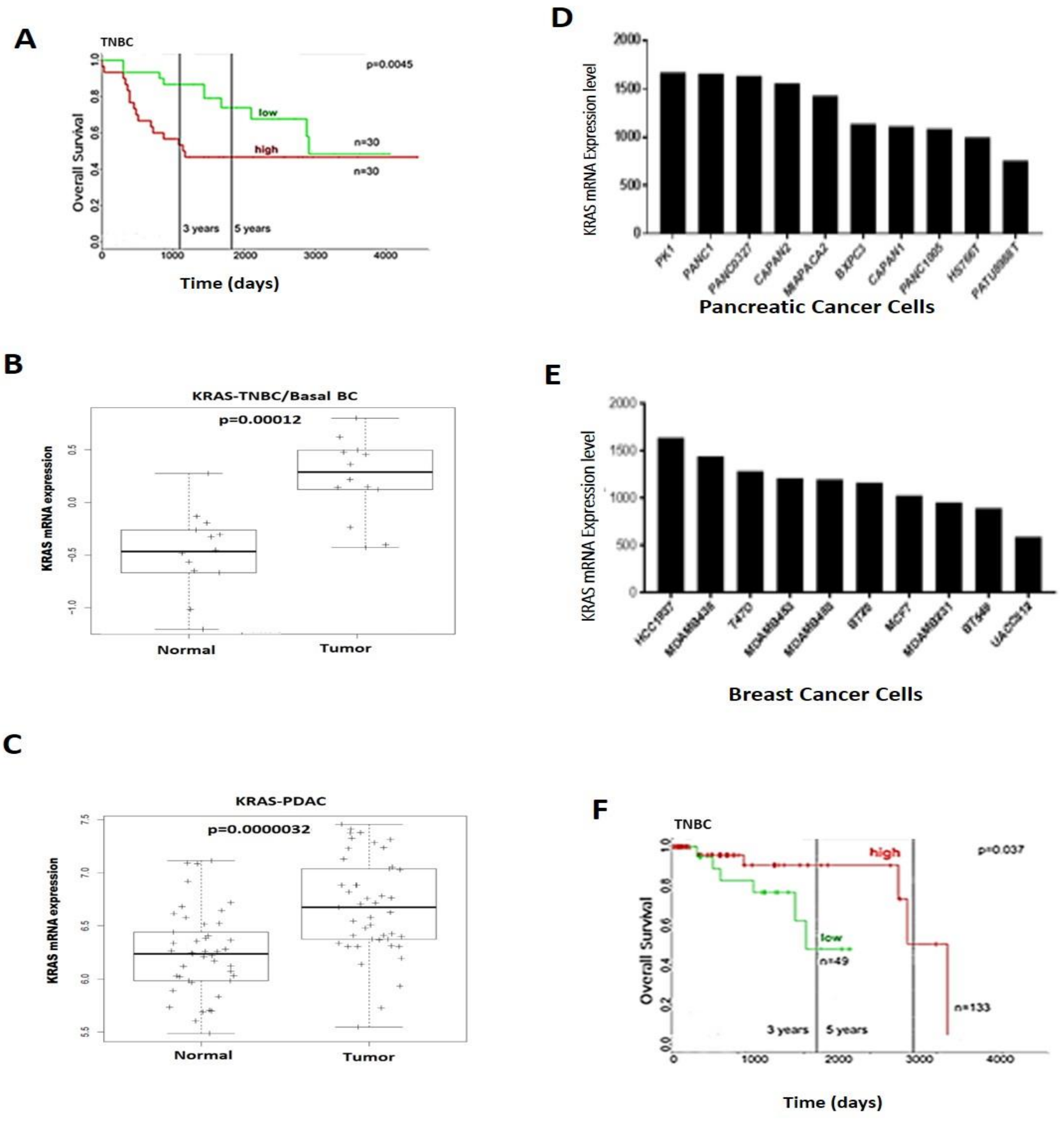

Supplementary Figure 1: KRAS is frequently upregulated in pancreatic and Breast cancer tissues. (A) Low KRAS mRNA expression is associated with high overall survival in patients with TNBC (n = 60, p = 0.0045). Data of TNBC were obtained from the PROGgeneV2 database. **(B)** The expression level of

KRAS mRNA in 45 pancreatic cancer tissues versus 45 normal tissues according to the GEO database (GSE28735), P=0.0000032. **(C)** The expression level of KRAS mRNA in 14 basal-like breast cancer tissues versus 14 normal breast tissues according to TCGA database, p=0.00012. **(D, E)** Analysis of Cancer Cell Line Encyclopedia (CCLE) database for KRAS expression profile in PDAC and BC cells. CCLE converted raw Affymetrix CEL files to a single value for each probeset using the robust multi-array average (RMA) and quantile normalization. PANC1, MiaPaCa-2, MDA-MB-231, and MDA-MB-436 cells are selected for further experiments based on the cell expression data. **(F)** High miR-873 expression is associated with high overall survival in patients with TNBC (n= 182, p = 0.037). Data of TNBC were obtained from miRpower tool (http://kmplot.com/analysis/).

Supplementary Fig. 2

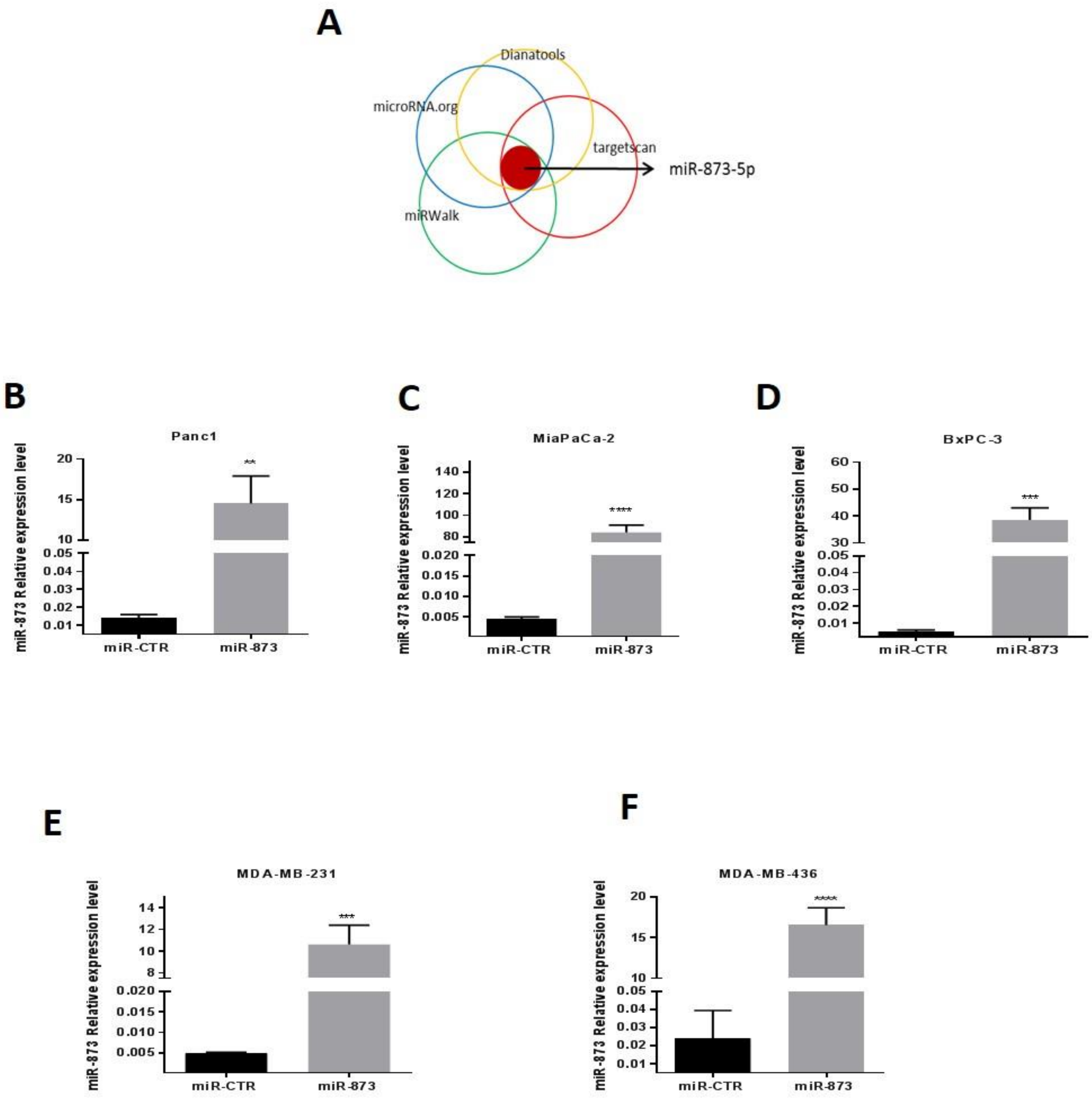

Supplementary Figure 2. Ectopic expression of miR-873 increases relative miR-873 expression levels in pancreatic ductal adenocarcinoma (PDAC) and triple-negative breast cancer (TNBC) cells. (A) Computational algorithms, including Targetscan, Diana tools microRNA.org and miRWalk2.0 that predict the potential of microRNAs binding to the 3′

untranslated region of KRAS mRNA. Among the potential target mRNAs, miR-873-5p was identified. **(B-F)** Cells were transfected with miR-873-100nM or control mimic for 72 h and miR-873 expression levels were analyzed in PDAC and TNBC cells using quantitative reverse-transcriptase polymerase chain reaction. Data represent means + standard error of three independent experiments. **p < 0.01; ***p < 0.001; ****p < 0.0001.

Supplementary Fig. 3

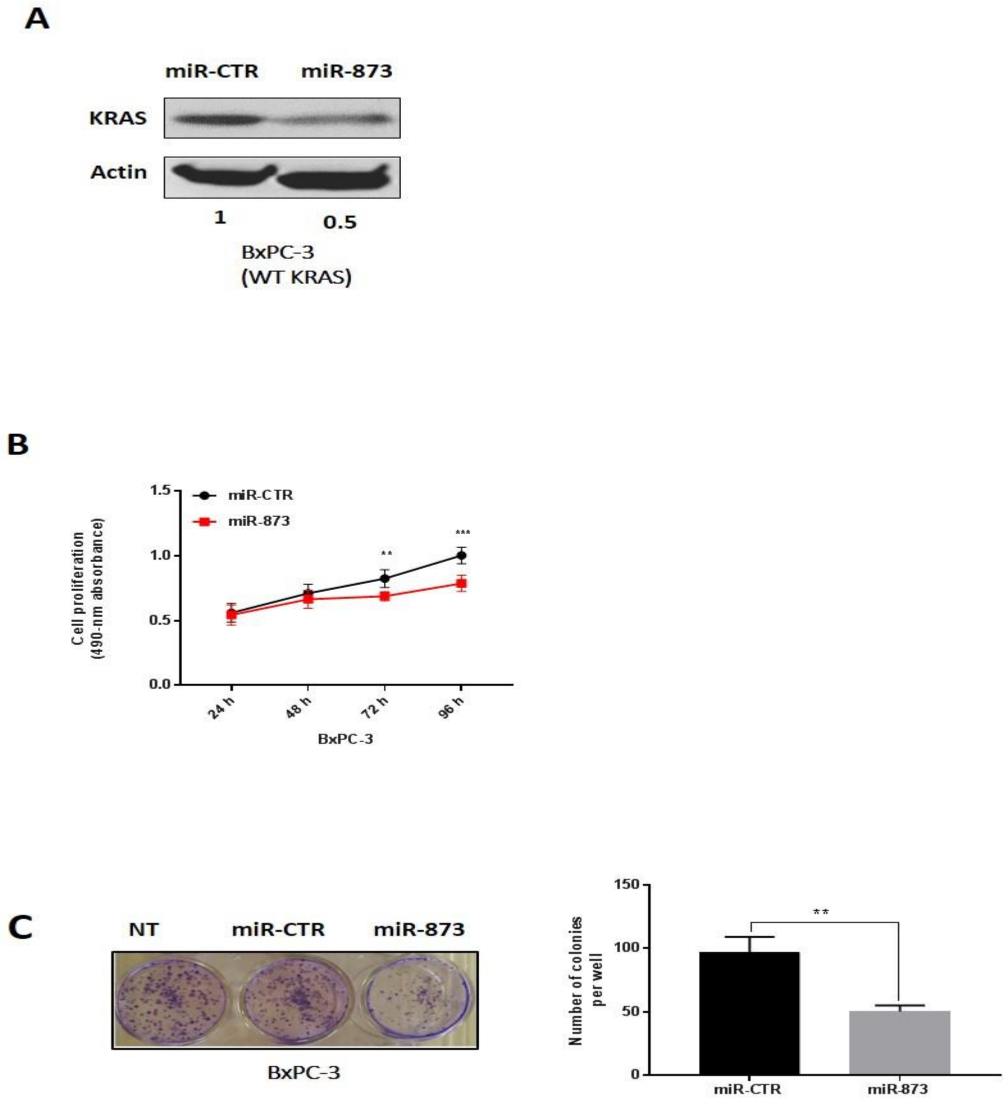

Supplementary Figure 3. Ectopic expression of miR-873 inhibits KRAS in pancreatic ductal adenocarcinoma cells harboring wild-type (WT) KRAS. (A) BxPC-3 cells were transfected with miR-873 or control mimic for 48 h and KRAS expression levels were analyzed by Western blot. (**B, C**) miR-873 suppresses cell proliferation (**B**) and colony formation (**C**) in BxPC-3 cells. Data represent means + standard error of three independent experiments. $^{**}p < 0.01$; $^{***}p < 0.001$ (compared with control group).

Supplementary Fig. 4

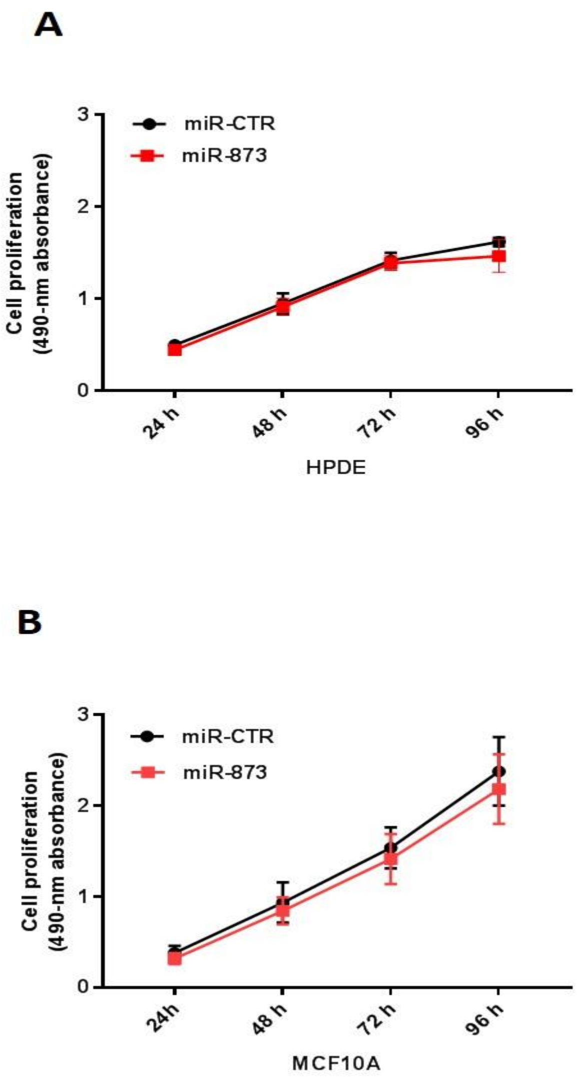

Supplementary Figure 4. MiR-873 has no remarkable effect on the proliferation of normal cells. The short-term effects of ectopic expression of miR-873 on the viability of normal pancreatic HPDE cells (**A**) and normal breast epithelial MCF10A cells (**B**) were examined using the MTS assay. The mean absorbance at 490 nm was determined at 48, 72, and 96 h. Data represent means + standard error of three independent experiments.

Supplementary Fig. 5

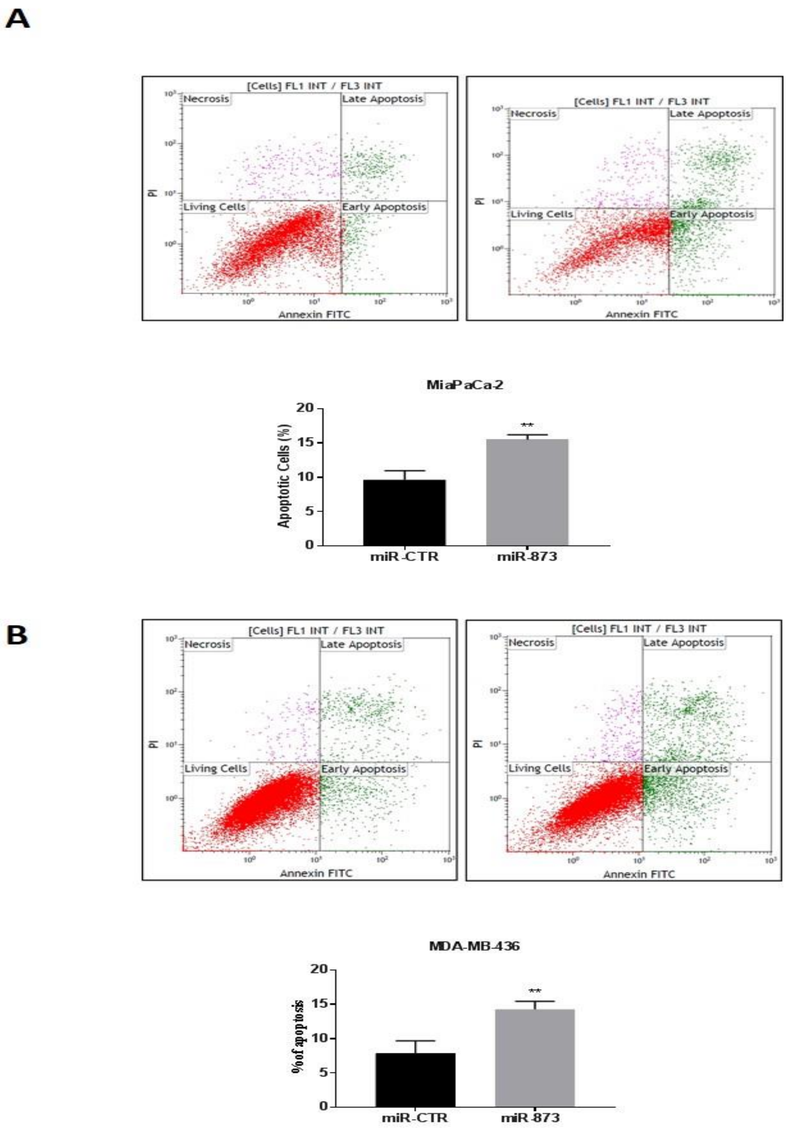

Supplementary Figure 5. MiR-873 triggers apoptosis in MiaPaCa-2 and MDA-MB-436 cells. miR-873 mimic or control mimic were transfected in MiaPaCa-2 **(A)** and MDA-MB-436 **(B)**, after 72h, cells were analyzed by Annexin V-FITC and PI double-staining and positive cells were detected and quantified by FACS analysis. The represented percentages show positive cells at both early and late apoptosis. Data are represented as mean ± SE. ** $P < 0.01$ indicates a significant difference compared with control group.

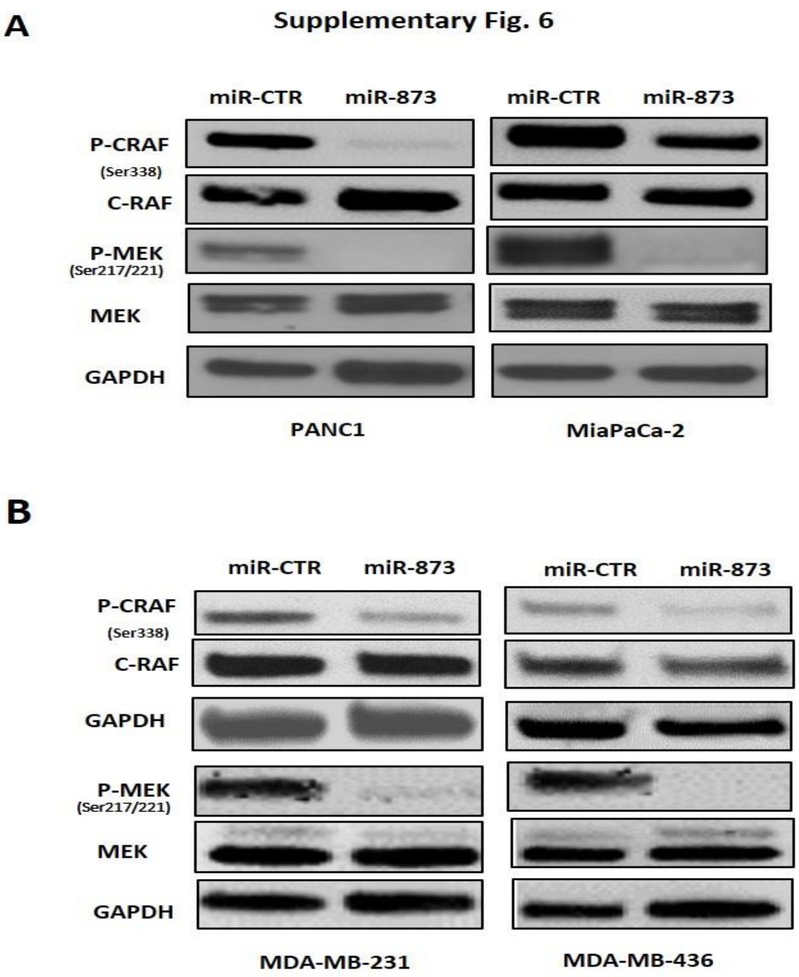

Supplementary Figure 6. MiR-873 inhibits KRAS downstream effectors.

Overexpression of miR-873 inhibited p-C-RAF and p-MEK1/2 in both PANC1, MiaPaCa-2 cells **(A)**, MDA-MB-231 and MDA-MB-436 **(B)**.

Supplementary Fig. 7

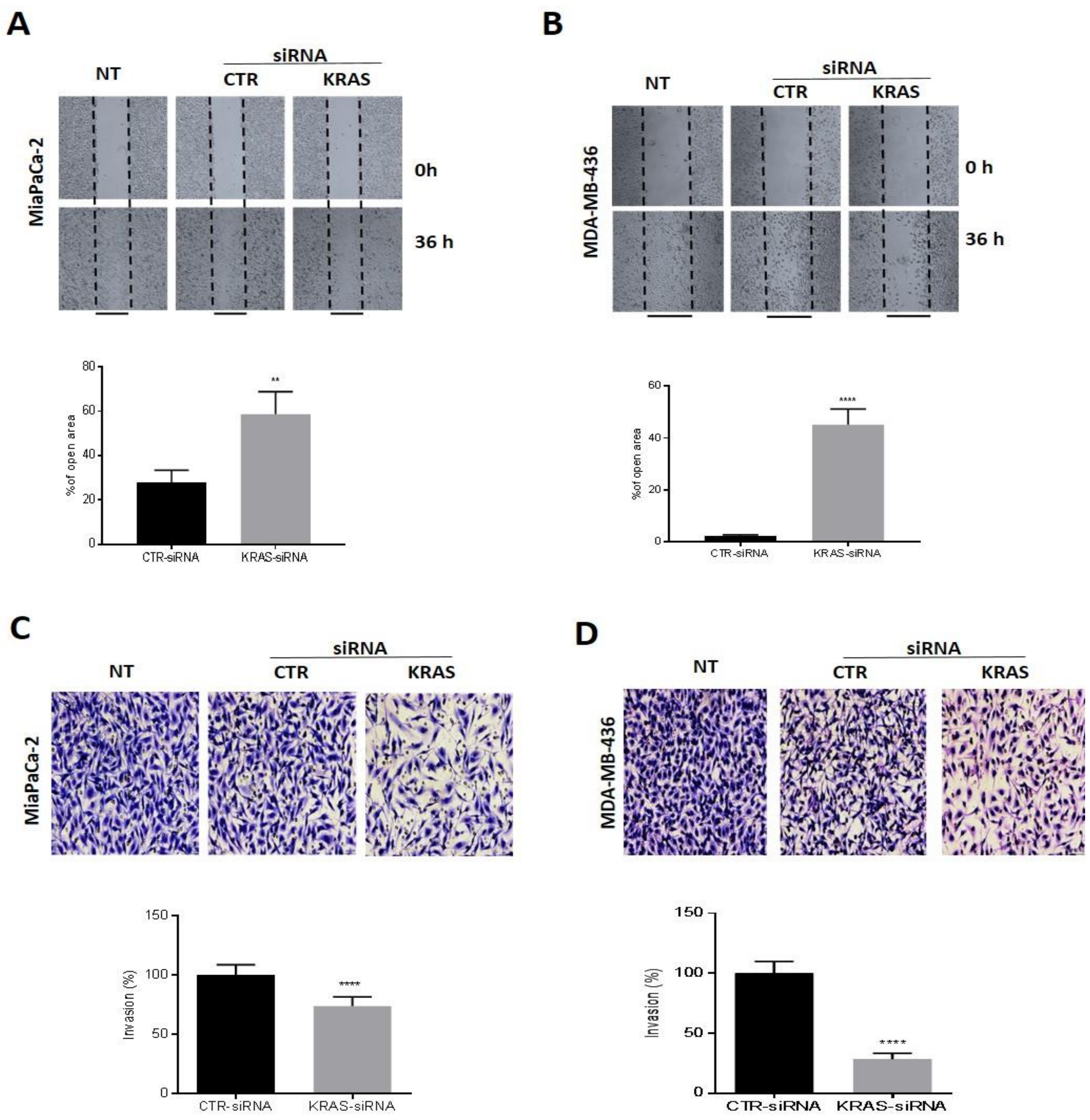

Supplementary Figure 7. Knockdown of KRAS by siRNA leads to inhibition of cell migration and invasion in vitro. Silencing of KRAS in PDAC (MiaPaCa-2) and TNBC (MDA-MB-436) cells led to a significant inhibition of cell migration **(A, B)** and invasion **(C, D)**. Data represent means + standard error of three independent experiments. **p < 0.01; ****p < 0.0001.

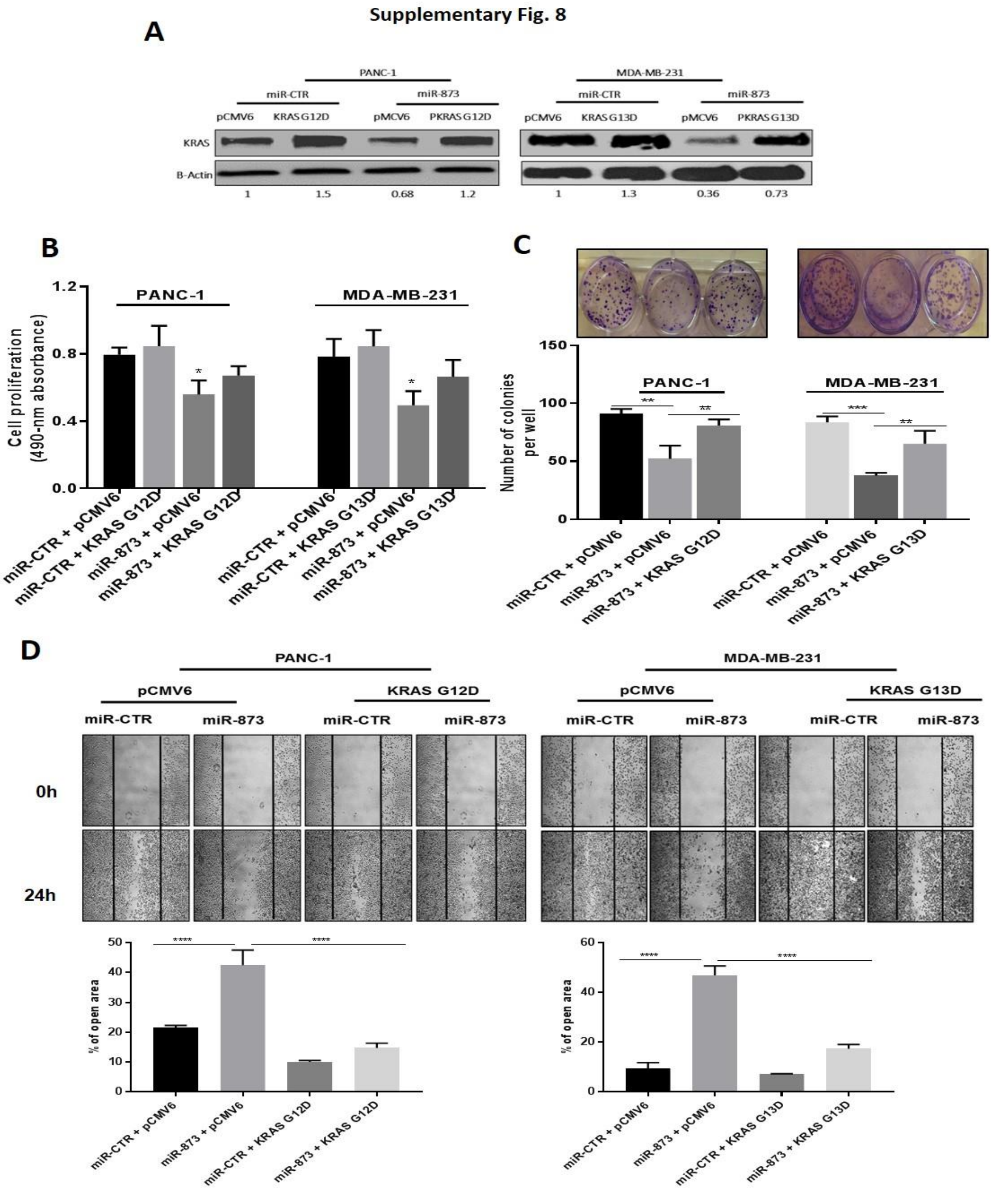

Supplementary Figure 8. Overexpression of KRAS rescues the effects of miR-873 on PDAC and TNBC cells. (A) Cells were transfected with the KRAS mutated clone vector -G12D for

PANC-1 and G13D for MDA-MB-231- and /or pMCV6 empty vector, then cells were transfected with miR-873 or control mimic and were analyzed by Western blot 48 h later for the expression of KRAS. β-actin was used as a loading control. (**B, C)** Overexpression of KRAS in PANC-1 and MDA-MB-231 cells reversed the effects of miR-873 mimic treatment on cell proliferation and clonogenic cell growth. **(D)** KRAS overexpression reversed the effects of miR-873-induced inhibition of cell migration in PANC-1 and MDA-MB-231 cells. Data represent means + standard error of three independent experiments. * $P < 0.05$ **$p < 0.01$; *** $P < 0.001$ ****$p < 0.0001$.

Supplementary Fig. 9

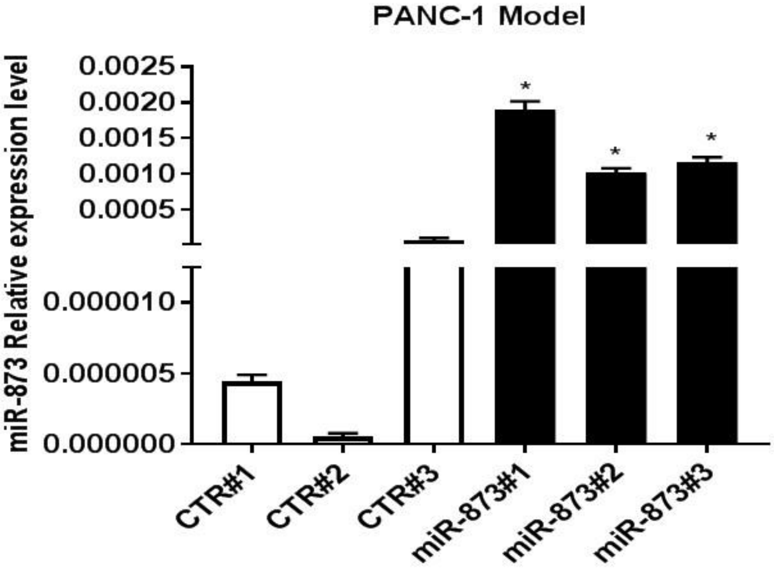

Supplementary Figure 9. Effects of in vivo treatment with nanoliposomal miR-873 on the weight of mice bearing pancreatic ductal adenocarcinoma (PDAC) and triple-negative breast cancer (TNBC) tumors. In mice with PDAC PANC1 **(A)** and TNBC MDA-MB-231 **(B)** xenografts, treatment with miR-873 for 4 to 5 weeks did not cause any change in the mean mouse weight after 4 weeks. Data represent means + standard error.

Supplementary Fig. 10

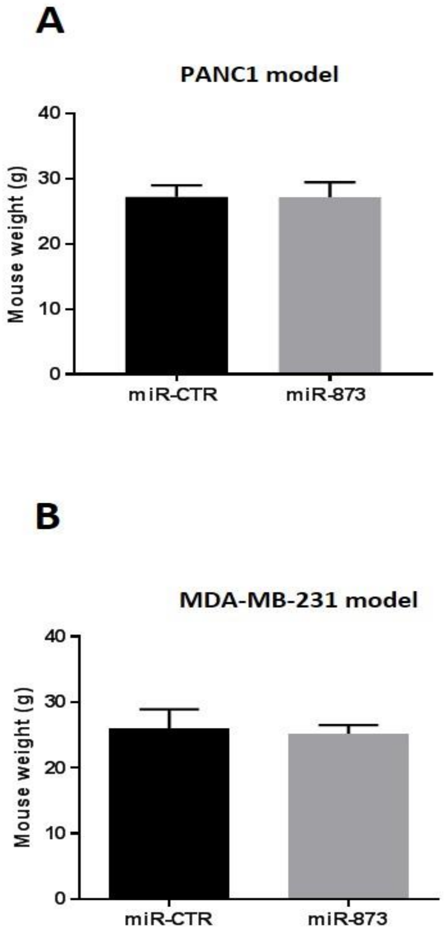

Supplementary Figure 10. Confirmation of successful delivery of miR-873 into tumor tissues. PANC-1 cells were injected into female nude mice (n=5). After tumor notice, mice were then treated with either miR-CTR or miR-873 mimic liposomal nano-particles intravenously once every 4 days, for 5 weeks. Tumor tissues were processed and total RNA was isolated from three random samples per each group and level of miR-873 was determined in those samples. U6 was utilized as an internal control. Mice treated with miR-873 showed higher expression levels of miR-873 than those treated with miR-CTR. Data represent means + standard error. * $P < 0.05$

Supplementary Fig. 11

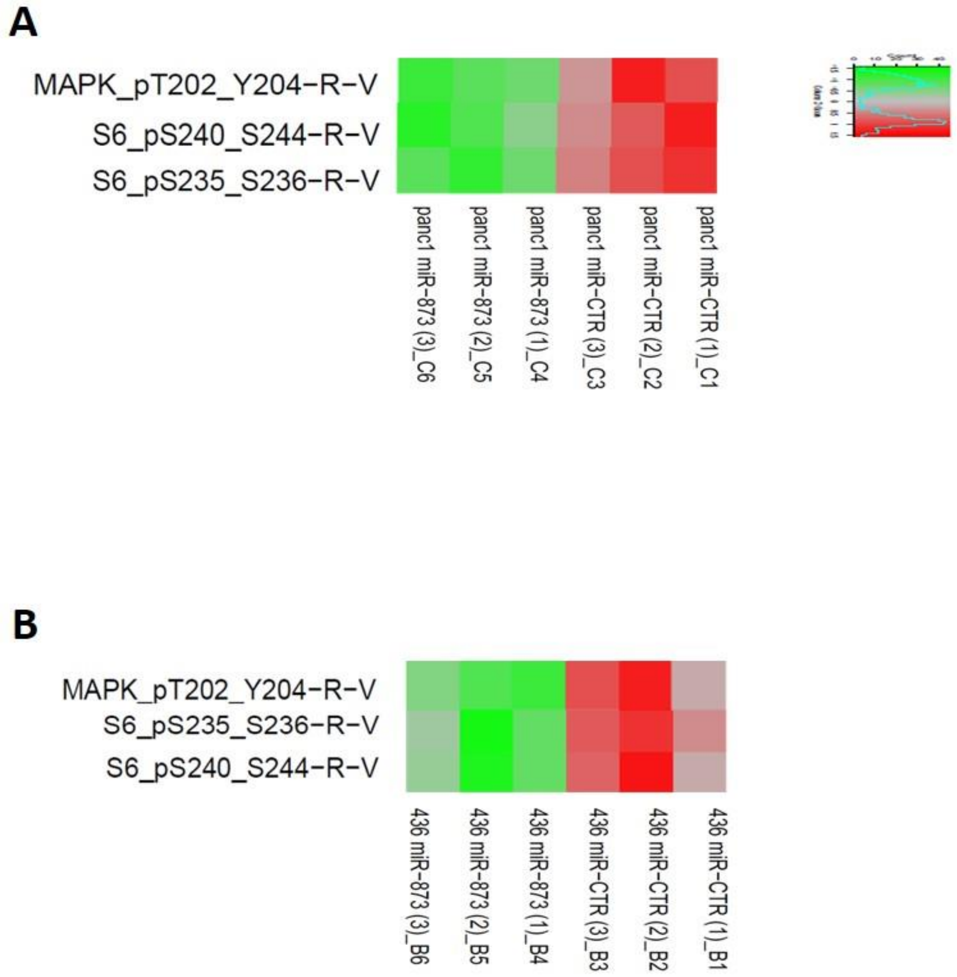

Supplementary Figure 11. Reverse phase protein array (RPPA) results reveal an array of altered proteins in PANC1 and MDA-MB-436 cells. (A, B) The heat map of RPPA analysis shows the results, with significant changes in cells after treatment with miR-873 and control miRNA. The expression ratios for a given sample group of interest were represented by their mean. Rows, proteins; columns, signal ratios of miR-873– or control miRNA–transfected PANC1 and MDA-MB-436 cells. For each protein, red indicates that the expression level of that protein was higher in miR-873–transfected cells than in control miRNA–transfected cells, and green indicates that the expression level was lower.

Supplementary Table 1: Median survival of pancreatic cancer patients and basal-like breast cancer patients.

Supplementary Table 1:

Pancreatic cancer		Median Survival
	Low KRAS	23.4
	High KRAS	18.7
	Low mir-873	19.7
	High mir-8A73	22.8
Basal like breast cancer		
	Low KRAS	115.7
	High KRAS	Undefined (>180)
	Low mir-873	Undefined (>55)
	High mir-873	115.7
