# Supplementary material for: The Modulatory Role of MicroRNA-873 in the Progression of KRAS-Driven Cancers
Source: Mol Ther Nucleic Acids. 2018 Dec 13;14:301–17. doi: 10.1016/j.omtn.2018.11.019 (PMC6348737; doi:10.1016/j.omtn.2018.11.019)
Supplement: Document S2. Article plus Supplemental Information [file mmc2.pdf]

Molecular Therapy
Nucleic Acids
Original Article

The Modulatory Role of MicroRNA-873 in the Progression of KRAS-Driven Cancers

Hamada A. Mokhlis,[1,2] Recep Bayraktar,[1] Nashwa N. Kabil,[1] Ayse Caner,[1] Nermin Kahraman,[1] Cristian Rodriguez-Aguayo,[1] Erika P. Zambalde,[1] Jianting Sheng,[3] Kübra Karagoz,[4] Pinar Kanlikilicer,[1] Abdel Aziz H. Abdel Aziz,[2] Tamer M. Abdelghany,[2] Ahmed A. Ashour,[2] Stephen Wong,[3] Michael L. Gatza,[4] George A. Calin,[1,5] Gabriel Lopez-Berestein,[1,5] and Bulent Ozpolat[1,5]

[1]Department of Experimental Therapeutics, The University of Texas MD Anderson Cancer Center, Houston, TX, USA; [2]Department of Pharmacology and Toxicology, Faculty of Pharmacy, The University of Al-Azhar, Cairo, Egypt; [3]Department of Systems Medicine & Bioengineering, Houston Methodist Institute for Academic Medicine Research Institute, Houston Methodist Weill Cornell Medical College, Houston, TX, USA; [4]Rutgers Cancer Institute of New Jersey, Rutgers, The State University of New Jersey, New Brunswick, NJ, USA; [5]Center for RNA Interference and Non-Coding RNAs, The University of Texas MD Anderson Cancer Center, Houston, TX, USA

KRAS is one of the most frequently mutated proto-oncogenes in pancreatic ductal adenocarcinoma (PDAC) and aberrantly activated in triple-negative breast cancer (TNBC). A profound role of microRNAs (miRNAs) in the pathogenesis of human cancer is being uncovered, including in cancer therapy. Using *in silico* prediction algorithms, we identified miR-873 as a potential regulator of KRAS, and we investigated its role in PDAC and TNBC. We found that reduced miR-873 expression is associated with shorter patient survival in both cancers. miR-873 expression is significantly repressed in PDAC and TNBC cell lines and inversely correlated with KRAS levels. We demonstrate that miR-873 directly bound to the 3′ UTR of KRAS mRNA and suppressed its expression. Notably, restoring miR-873 expression induced apoptosis; recapitulated the effects of KRAS inhibition on cell proliferation, colony formation, and invasion; and suppressed the activity of ERK and PI3K/AKT, while overexpression of KRAS rescued the effects mediated by miR-873. Moreover, *in vivo* delivery of miR-873 nanoparticles inhibited KRAS expression and tumor growth in PDAC and TNBC tumor models. In conclusion, we provide the first evidence that miR-873 acts as a tumor suppressor by targeting KRAS and that miR-873-based gene therapy may be a therapeutic strategy in PDAC and TNBC.

INTRODUCTION

The rat sarcoma (RAS) genes (*KRAS*, *HRAS*, *NRAS*, and *MRAS*) represent a family of small GTPases that are activated indirectly via external stimuli, such as ligand-dependent activation of receptor tyrosine kinases.[1,2] KRAS (Kirsten-rat sarcoma viral oncogene homolog) was the first RAS family protein identified. It primarily functions as a critical on/off switch in cell-signaling networks that connects upstream extracellular signals to downstream pathways to the nucleus.[3] Signals emanating from RAS proteins are relayed through proto-oncogene serine/threonine-protein kinase (RAF), mitogen-activated protein kinase (MAPK), and extracellular signal-regulated kinase (ERK)1/2 (also known as MAPK1/3) to the nucleus, where downstream transcription factors, including ETS-1, ETS-2, ELK-1, FOS, and MYC, drive transcriptional programs of cell proliferation and survival, including migration, invasion, cytoskeletal changes, and the cell cycle.[4–6]

Activating *KRAS* gene mutations occur in one-third of human cancers, including adenocarcinomas of the pancreas (80%–90%), colon (45%), and lung (30%–50%),[7] and also in biliary tract malignancies, endometrial cancer, cervical cancer, bladder cancer, liver cancer, myeloid leukemia,[8,9] and breast cancer.[10] Although canonical mutations in the KRAS pathway are uncommon (∼5%–12%),[11,12] overexpression of KRAS[13] and transcriptional signatures of activation of the KRAS/MAPK pathway are frequently observed in breast cancer cells,[14] often accompanied by epidermal growth factor receptor (EGFR) mutations or amplifications.[15,16] Mounting evidence suggests that the KRAS/MAPK pathway is highly prevalent and constitutes a major component of oncogenic activity in triple-negative breast cancer (TNBC), more so than in other subtypes of breast cancer.[14,17–19] Owing to the failure of farnesyltransferase inhibitors in clinical trials and the lack of small molecule therapeutics approved for directly targeting KRAS,[20] current strategies involve targeting downstream components in the pathway, such as mitogen-activated protein kinase kinase (MEK) and phosphatidylinositol 3-kinase (PI3K) inhibitors.[5]

Non-coding RNAs (ncRNAs) such as microRNAs (miRNAs) are small non-coding RNAs (∼22 nt) that regulate gene expression at

Received 13 July 2018; accepted 29 November 2018;
https://doi.org/10.1016/j.omtn.2018.11.019.

Correspondence: Bulent Ozpolat, MD, PhD, Department of Experimental Therapeutics, Unit 422, The University of Texas MD Anderson Cancer Center, 1515 Holcombe Boulevard, Houston, TX 77030, USA.
E-mail: bozpolat@mdanderson.org

This is an open access article under the CC BY license (http://creativecommons.org/licenses/by/4.0/).

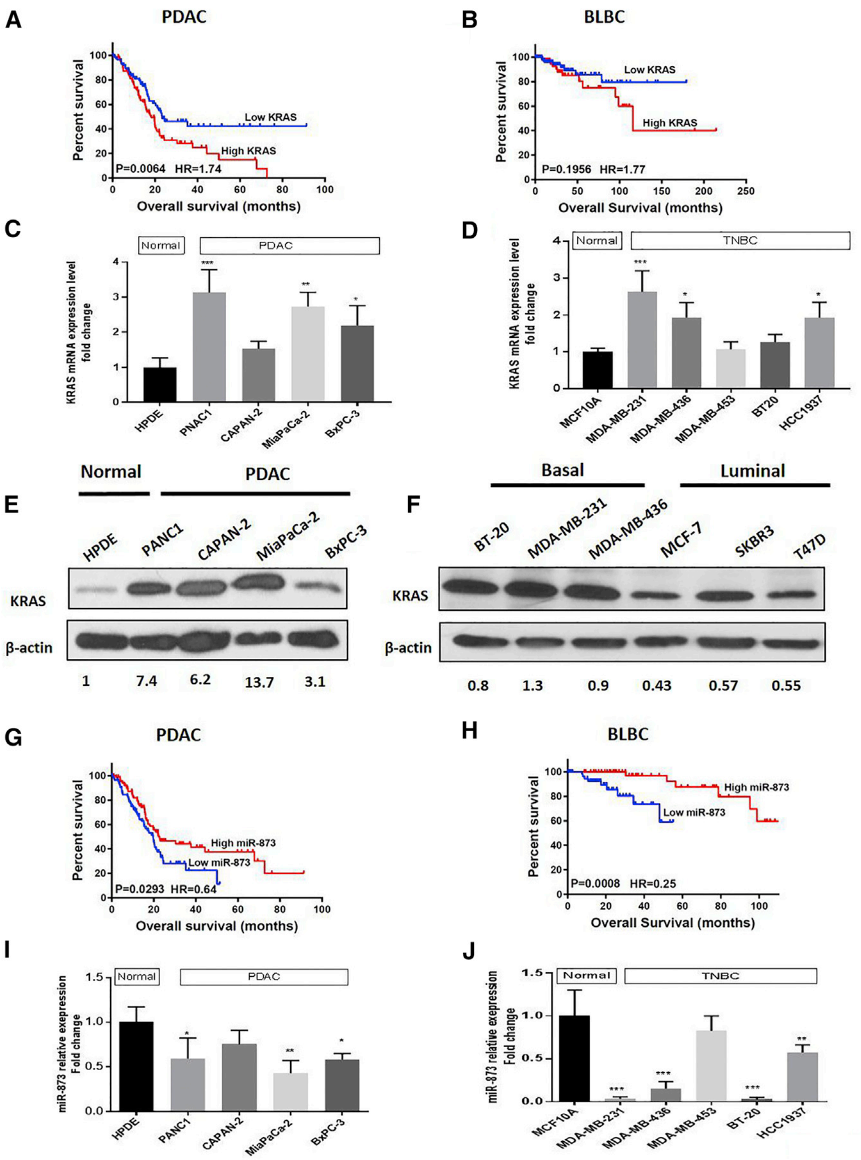

Figure 1. Increased KRAS and Reduced miR-873 Expression Levels Are Associated with Poor Overall Survival in Patients with PDAC and BLBC

(A and B) High KRAS mRNA expression is associated with poor overall survival in patients with PDAC (A; n = 177, p = 0.0064), BLBC (B; n = 172, p = 0.1956), according to Kaplan-Meier survival analysis. The number of patients at risk in the low and high KRAS groups at different time points is presented inside the graph. (C and D) Expression levels of KRAS mRNA in PDAC (C) and TNBC (D) cell lines were determined by qRT-PCR. Data were normalized to the expression of GAPDH and represent means + SE of three independent experiments. *p < 0.05, **p < 0.01, ***p < 0.001. (E) Most of PDAC cells have a high Kras protein expression level compared to normal HPDE cells. (F) KRAS protein expression is activated in basal-like triple-negative breast cancer cells compared to luminal types. Reduced expression of miR-873 is associated with poor overall survival in patients with PDAC (G; n = 177, p = 0.0293) and BLBC (H; n = 118, p = 0.0008), as determined by Kaplan-Meier analysis. The number of patients at risk in the low and high miR-873 groups at different time points is presented inside the graph. (I and J) Expression levels of miR-873 in PDAC (I) and TNBC (J) cell lines and normal pancreatic ductal epithelium and normal breast epithelial MCF10A cells were determined by qRT-PCR. Data were normalized to the expression of GAPDH and represent means + SE of three independent experiments. *p < 0.05, **p < 0.01, ***p < 0.001.

the posttranscriptional level.[21] miRNAs bind to the 3′ UTR of their protein-target genes (mRNAs), and they suppress protein translation by either blocking the initiation of translation or accelerating the degradation of the target mRNAs. Since miRNAs were first discovered more than two decades ago, about 60% of all human protein-coding genes are known to be the direct targets of miRNAs.[22] Recent studies have indicated that many miRNAs are aberrantly expressed in tumor cells and contribute to tumorigenesis and tumor progression by regulating signaling pathways, apoptosis, angiogenesis, the cell cycle, senescence, migration, and metastasis.[23–25]

In this study, we found that expression of miR-873 was reduced in pancreatic ductal adenocarcinoma (PDAC) and TNBC cells and associated with significantly longer patient survival, indicating a tumor suppressor function in pancreatic and breast cancer patients. At the molecular level, our findings elucidate the role of miR-873 in targeting KRAS, which critically controls PDAC and TNBC progression. Meanwhile, miR-873 inversely correlates with KRAS expression, which also is associated with shorter patient survival. Restoration of miR-873 expression of PDAC and TNBC models suppressed cell proliferation, migration, invasion, and tumorigenesis by inhibiting the KRAS/ERK and KRAS/PI3K axes. Overall, *in vivo* therapeutic delivery of miR-873 could be a potential novel therapeutic strategy to control KRAS signaling in PDAC and TNBC.

RESULTS

Increased KRAS Levels Are Associated with Poor Clinical Outcomes in Patients with PDAC and TNBC

To explore the clinical significance of *KRAS* expression, we analyzed a subset of patients with PDAC and basal-like breast cancer (BLBC) from The Cancer Genome Atlas (TCGA) by the Kaplan-Meier method, and also, we used the PROGgeneV2 tool[26] incorporating survival data associated with KRAS in patients with TNBC. Patients with high KRAS expression had significantly lower overall survival rates than did patients with low expression (PDAC: n = 177, p = 0.0064; BLBC: n = 172, p = 0.1956; Figures 1A and 1B; TNBC: n = 60, p = 0.0045; Figure S1A).

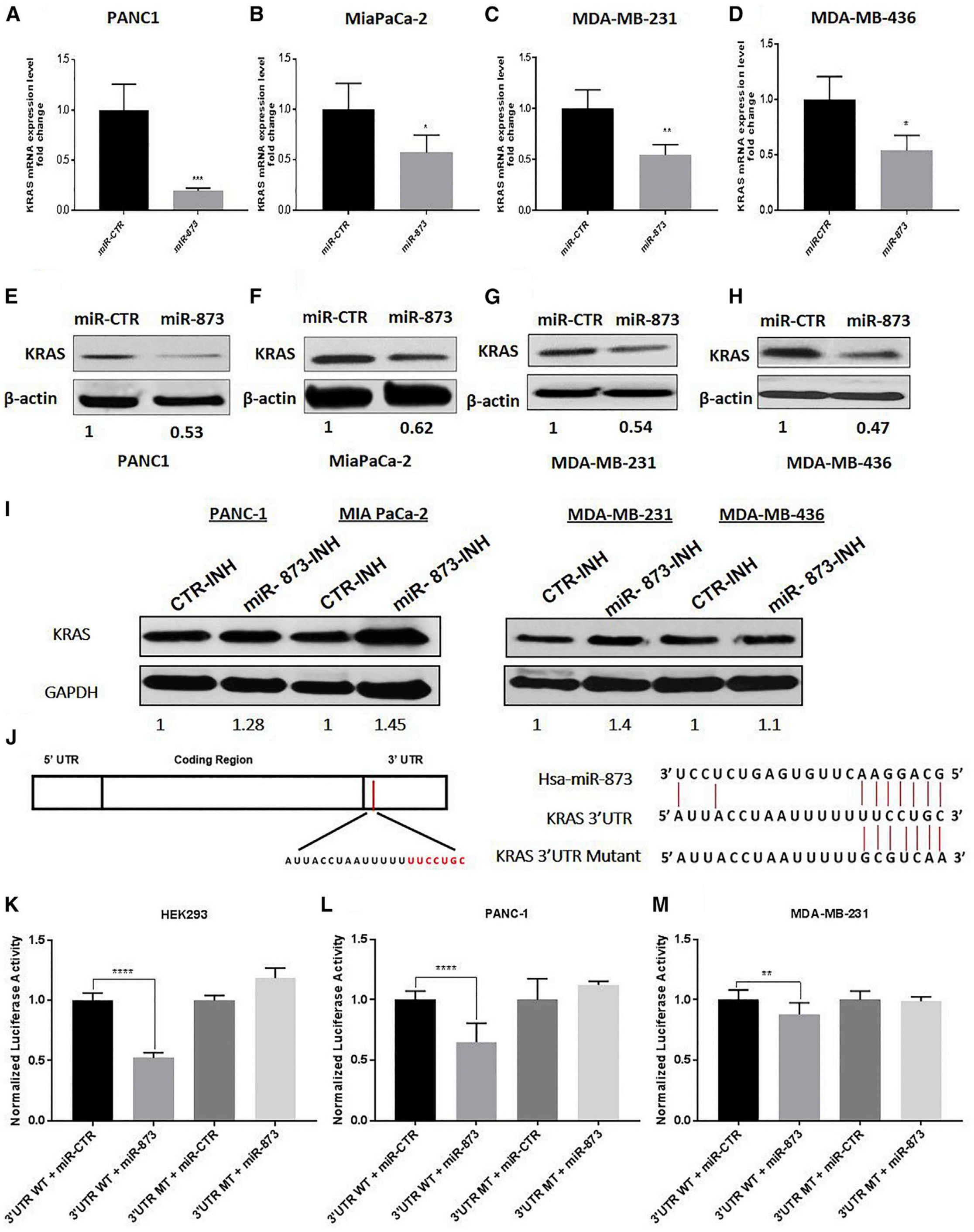

Figure 2. miR-873 Directly Binds to the 3′ UTR of KRAS mRNA and Suppresses Its Expression in PDAC and TNBC Cells

(A–H) Ectopic expression of miR-873 in PDAC (A and E, PANC1; and B and F, MiaPaCa-2) and TNBC (C and G, MDA-MB-231; and D and H, MDA-MB-436) cells led to decreased KRAS mRNA expression levels, according to qRT-PCR (A–D), and decreased KRAS protein expression levels, according to western blot analysis (E–H). Data represent means + SE of three independent experiments. *$p < 0.05$, **$p < 0.01$, ***$p < 0.001$. (I) The effects of miR-873 inhibitor on KRAS protein overexpression. Cells were transfected with miR-873 inhibitor or control inhibitor for 72 h, and KRAS protein expression levels were analyzed by western blotting. (J) The predicted binding site of

(legend continued on next page)

To identify the differentially expressed KRAS mRNA in pancreatic tissues, we found that KRAS mRNA was significantly upregulated in human pancreatic tumor tissues (n = 45) compared to normal tissues (n = 45) (p = 3.26e−06) according to the GEO database (GEO: GSE28735) (Figure S1B). At the same time, its expression was markedly high in BLBC tissues (n = 14) with respect to normal breast tissues (n = 14) (p = 0.00012), according to TCGA database (Figure S1C). qRT-PCR analysis showed that the *KRAS* gene was significantly upregulated in most PDAC and TNBC cells ($p < 0.05$; Figures 1C and 1D). Meanwhile, basal expression of KRAS mRNA was checked in a pancreatic and breast cancer cell panel according to the data from cancer cell line Encyclopedia (CCLE) (Figures S1D and S1E). The median survival for each set is included in Table S1. On the protein level, we noticed marked upregulation of KRAS protein expression level in most of pancreatic cancer cells in comparison to normal pancreatic cells (Figure 1E). Meanwhile, malignant basal-type (MDA-MB231, MDA-MB-436, and BT20) breast cancer cells had a higher expression level of KRAS compared with luminal-type breast cancer cell lines (MCF7, SKBR3, and T47D; Figure 1F).

Reduced miR-873 Expression Is Associated with Poor Overall Survival in Patients with PDAC and TNBC

To identify clinically relevant miRNAs that bind and regulate the *KRAS* gene, we first used computational algorithms, including TargetScan,[27] Diana tools,[28] microRNA.org,[29] and miRWalk2.0,[30] which predict the potential of miRNAs targeting the 3′ UTR of KRAS mRNA. Among the potential miRNAs, miR-873-5p was identified owing to its significance in overall survival in patients with PDAC and TNBC (Figure S2A). To elucidate the clinical significance of miR-873 expression, we analyzed a subset of patients with PDAC and BLBC from Kaplan-Meier data, and also, we used the miRpower tool (http://kmplot.com/analysis/) to analyze survival data associated with miR-873 in patients with TNBC. The overall survival rate was dramatically higher in patients with high miR-873 expression than in patients with low miR-873 expression (PDAC: n = 177, p = 0.0293; Figure 1G; BLBC: n = 118, p = 0.0008; Figure 1H; TNBC: n = 182, p = 0.037; Figure S1F). The median survival for each set is provided in Table S1.

miR-873 Expression Is Reduced in PDAC and TNBC Cell Lines

Next, we analyzed the basal expression level of miR-873 in PDAC cell lines (PANC1, Capan-2, MiaPaCa-2, and BxPC-3) and TNBC cell lines (MDA-MB-231, MDA-MB-436, MDA-MB-453, BT-20, and HCC1937) using qRT-PCR, and we compared it with that in normal human pancreatic ductal epithelial (HPDE) cells and normal human mammary epithelial cells (MCF10A). Our results showed that basal miR-873 expression was significantly lower in most PDAC and TNBC cell lines (Figures 1I and 1J) compared with normal cells. We selected KRAS-mutated PANC1 and MiaPaCa-2 and wild-type KRAS BxPC-3 pancreatic cells and MDA-MB-231 (KRAS-mutated) and MDA-MB-436 (wild-type KRAS) TNBC cells for further studies.

miR-873 Directly Binds to the 3′ UTR of KRAS mRNA to Regulate Its Expression

To examine the role of miR-873 in *KRAS* gene expression, cells were transfected with miR-873 mimic or control mimic oligonucleotides and analyzed at 48 h. miR-873 transfection led to significantly higher expression of miR-873 compared with control miRNA (Figures S2B–S2F), and it reduced KRAS mRNA and protein expression in PDAC cells (PANC1 and MiaPaCa-2) and TNBC cells (MDA-MB-231 and MDA-MB-436; Figures 2A–2H), as well as BxPC-3 cells with wild-type KRAS (Figure S3A). On the other hand, the blocking of miR-873 with antimiR had a reverse effect on the protein expression of KRAS (Figure 2I).

Next, we examined the direct role of miR-873 in posttranscriptional regulation of *KRAS* gene expression. Using online prediction tools, we identified the consensus sequences on the 3′ UTR of KRAS mRNA (Figure 2J), and we performed luciferase reporter assays to show that miR-873 directly binds to KRAS mRNA in its 3′ UTR. The binding site for miR-873 in the *KRAS* 3′ UTR was cloned into a pEZX-MT06 miRNA reporter vector that also contains the luciferase gene (pEZX-MT06-3′ UTR). The resulting plasmids were co-transfected into HEK293, PANC1, and MDA-MB-231 cells along with the miR-873 or the miRNA control. Luciferase activity was measured 48 h after transfection. We found that the luciferase expression was significantly decreased in cells that were co-transfected with miR-873 and the KRAS 3′ UTR compared with the controls (Figures 2K–2M). However, no decrease was observed in cells that were co-transfected with miR-873 and the mutant KRAS 3′ UTR compared with the controls (Figures 2K–2M).

miR-873 Suppresses Proliferation and Colony Formation of PDAC and TNBC Cells

To examine the short-term effects of miR-873 on cell growth, we performed the MTS (3-(4,5-dimethylthiazol-2-yl)-5-(3-carboxymethoxyphenyl)-2-(4-sulfophenyl)-2H-tetrazolium) assay in PDAC and TNBC cells for 24, 48, 72, and 96 h. miR-873 overexpression led to a significant inhibition of proliferation of PANC1, MiaPaCa-2, MDA-MB-231, and MDA-MB-436 cells ($p < 0.05$; Figures 3A–3D). A similar inhibition effect was obtained in BxPC-3 cells after miR-873 treatment (Figures S3B and S3C). Moreover, ectopic expression of miR-873 did not result in any inhibition of cell viability in normal pancreatic epithelial and breast cells (HPDE and MCF10A) that were examined using the MTS assay (Figures S4A and S4B).

miR-873 in the 3′ UTR of human wild-type *KRAS* and sequences were determined. Mutations in the seed sequence of the full-length *KRAS* 3′ UTR are also shown. (K–M) Luciferase reporter assay results show that miR-873 directly targets the *KRAS* 3′ UTR-luciferase reporter (wild-type binding site) in HEK293 (K), PANC1 (L), and MDA-MB-231 (M) cells. The firefly luciferase activity of the reporter was normalized to the internal *Renilla* luciferase activity. Data represent means + SE of three independent experiments. **$p < 0.01$, ****$p < 0.0001$.

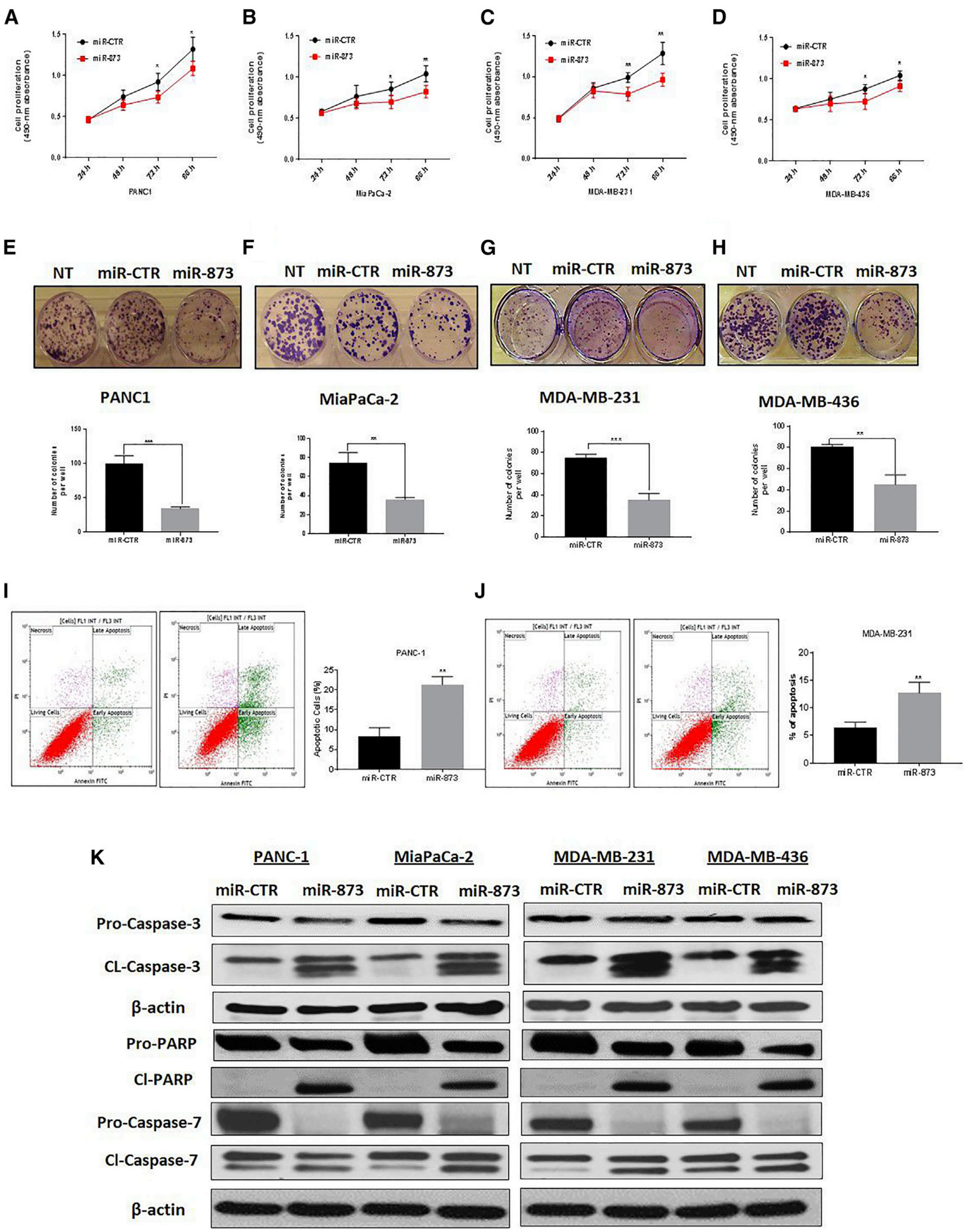

Figure 3. miR-873 Expression Suppresses Proliferation and Colony Formation and Triggers Apoptosis of PDAC and TNBC Cells

(A–D) The short-term effects of ectopic expression of miR-873 on the proliferation of PDAC (A, PANC1; and B, MiaPaCa-2) and TNBC (C, MDA-MB-231; and D, MDA-MB-436) cells were examined using the MTS assay, and the mean absorbance at 490 nm was determined at 48, 72, and 96 h. Data represent means + SE of three independent experiments. *p < 0.05, **p < 0.01. (E–H) The effects of expression of miR-873 on the clonogenic ability of PDAC (E, PANC-1; F, MiaPaCa-2) and TNBC (G, MDA-MB-23; H, MDA-MB-436) cells were determined by a colony formation assay. Upper panels (E–H) are representative images from the colony formation assay and lower panels represent quantification of the number of colonies formed. Data represent means + SE of three independent experiments. **p < 0.01, ***p < 0.001. (I and J) Ectopic expression of miR-873 triggered apoptosis in PANC1 (I) and MDA-MB-231 (J) cells. Cells were transfected with miR-873 or control mimic and analyzed by Annexin V-FITC

(legend continued on next page)

We further examined the effects of miR-873 on PDAC and TNBC clonogenicity using a colony formation assay. miR-873 expression significantly decreased colony formation in PANC1, MiaPaCa-2, MDA-MB-231, and MDA-MB-436 cells ($p < 0.05$) compared with untreated or control miRNA-transfected cells (Figures 3E–3H).

Ectopic Expression of miR-873 Induces Cell Apoptosis by Regulating Caspase-Dependent Apoptosis Pathways

Inhibition of cell growth in cancer cells is usually associated with concomitant activation of cell death pathways. We, therefore, examined the contribution of apoptosis to growth inhibition mediated by miR- 873 overexpression. We evaluated the rate of cellular apoptosis using Annexin V and propidium iodide (PI) staining for flow cytometry. The number of both early and late apoptotic PANC1, MIA PaCa-2, MDA-MB-231, and MDA-MB-436 cells at 72 h post-transfection of miR-873 was substantially higher, by ∼2.5-, ∼1.5-, ∼2-, and ∼2-fold, respectively, than the number of control miRNA-transfected cells (Figures 3I and 3J; Figures S5A and S5B). The induction of apoptosis was further confirmed in PDAC and TNBC cells by the expression of apoptosis-related proteins, including caspase-3, poly ADP ribose polymerase (PARP), and caspase-7, on western blot (Figure 3K).

miR-873 Suppresses Migration and Invasion in PDAC and TNBC Cells

To investigate the effect of miR-873 on migration, we performed the scratch wound-healing assay. As shown in Figures 4A–4D, the wound distance at different time points after miR-873 transfection was significantly larger than that of the control group in all cells, indicating that miR-873 expression leads to reduced cell motility and migration of PANC1 ($p = 0.0033$), MiaPaCa-2 ($p = 0.0001$), MDA-MB-231 ($p = 0.0003$), and MDA-MB-436 cells ($p = 0.0001$).

To elucidate the role of miR-873 expression in regulating PDAC and TNBC cell invasion, we performed the *in vitro* matrigel invasion assay after transfection with miR-873 mimic and control miRNA. Ectopic overexpression of miR-873 significantly reduced the number of invading PANC1, MiaPaCa-2, MDA-MB-231, and MDA-MB-436 cells by 78.42%, 85.72%, 83.62%, and 83.08%, respectively, in comparison with miR-control (Figures 4E–4H). Together, these results suggest that miR-873 expression suppresses invasion in both PDAC and TNBC cells.

miR-873 Inhibits Pathways Downstream of KRAS

KRAS signaling leads to the induction of clinically significant downstream pathways, including protein kinase B (AKT) and ERK, which are currently being targeted by small molecularly targeted therapeutics owing to the lack of approved KRAS inhibitors.[31,32] To determine whether miR-873-induced inhibition of KRAS signaling reduces these signaling pathways in PDAC and TNBC cells, we expressed miR-873 in the related cell lines, and we examined the activity of these pathways. Expression of miR-873 in PANC1, MiaPaCa-2, MDA-MB-231, and MDA-MB-436 cells markedly decreased the phosphorylation levels of p-AKT$^{(Ser473)}$ and p-ERK$^{(Thr202/Thy204)}$ (Figure 4I), p-C-RAF$^{(Ser338)}$, C-RAF, p-MEK1/2$^{(Ser217/221)}$, and MEK (Figures S6A and S6B).

Knockdown of KRAS Inhibited Cell Proliferation, Migration, and Invasion in PDAC and TNBC Cells

To determine whether the regulatory effects of miR-873 on proliferation, invasion, and migration in PDAC and TNBC cells are mediated by KRAS, we used small interfering RNA (siRNA)-mediated KRAS inhibition to recapitulate the tumor suppressor effects of miR-873 in PDAC and TNBC cells. KRAS knockdown significantly inhibited colony formation (Figures 5A–5D) and suppressed cell migration and invasion in both PDAC and TNBC cells (Figures 5E–5H; Figures S7A–S7D). siRNA-mediated suppression of KRAS and its downstream genes was confirmed by western blot analysis (Figure 5I). These results were similar to the effects of miR-873 overexpression.

KRAS Overexpression Reverses the miR-873-Mediated Effects

To further confirm that miR-873 mediates its inhibitory effects through downregulation of KRAS, we performed rescue experiments using mutated KRAS clone constructs to overexpress KRAS protein in PANC1 and MDA-MB-231 cancer cells. KRAS overexpression reversed the effects of miR-873 in downregulating KRAS (Figure S8A). Ectopic expression of KRAS plasmid rescued the suppressive effects of miR-873 on cell proliferation and colony formation in both cancer cell lines (Figures S8B and S8C). As expected, transfecting cells with miR-873 mimic decreased the migration ability of cells compared to control (CTR) miRNA-transfected cells. However, this effect was reversed in KRAS-overexpressed cells (Figure S8D).

In Vivo Therapeutic Administration of miR-873 by Nanoparticles Inhibits Tumor Growth in PDAC and TNBC Murine Models with Human Cancer Cell Xenografts

To determine the *in vivo* effects of miR-873 expression on PDAC and TNBC tumorigenesis, as well as the therapeutic potential of delivery of this miRNA, we systemically (intravenously) delivered miR-873 in PANC1, MiaPaCa-2, MDA-MB-231, and MDA-MB-436 orthotopic xenograft mouse models. Mice treated with miR-873 showed significantly less tumor growth than the control mice (Figures 6A, 6B, 7A, and 7B).

To test that our miRNA was delivered effectively to tumor tissues, we did qRT-PCR in PANC1 tumor tissues. Mice treated with miR-873 showed a marked increase in miR-873 expression level compared to those treated with miR-control (Figure S9). Tumor tissues were also

and PI double staining, and positive cells were detected and quantified by fluorescence-activated cell sorting (FACS) analysis. The represented percentages show positive cells at both early and late apoptosis. Data are represented as mean ± SE. **$p < 0.01$ indicates a significant difference compared with the control group. All experiments were independently performed three times. (K) Apoptotic-related protein expression was detected by western blot. miR-873-induced apoptosis was manifested after 72 h of transfection by an increase in cleavage of caspase-3, caspase-7, and PARP. β-actin was used as a loading control.

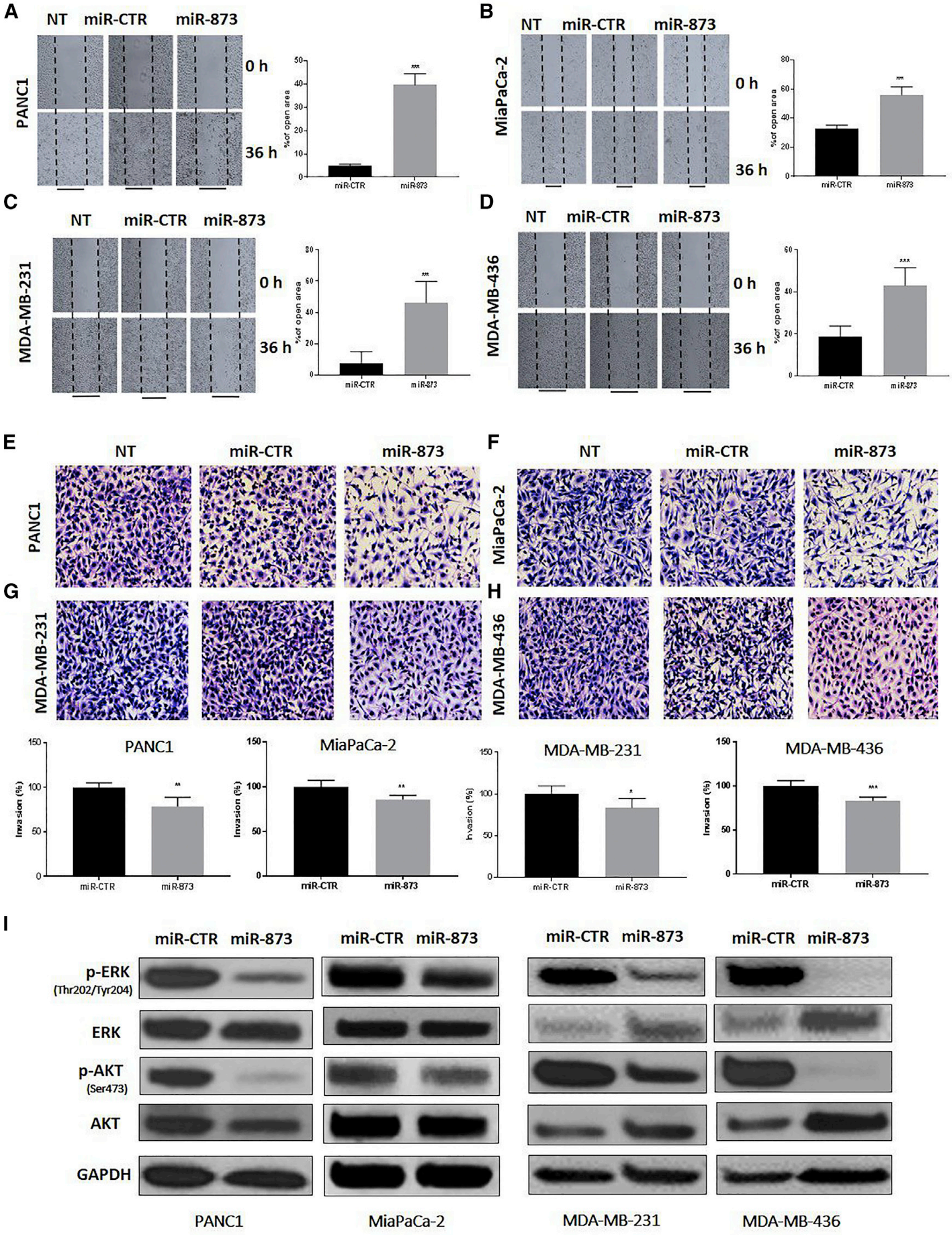

Figure 4. miR-873 Expression Inhibits Migration and Invasion Ability and Clinically Significant Pathways of PDAC and TNBC Cells

(A–H) PANC1 (A and E), MiaPaCa-2 (B and F), MDA-MB-231 (C and G), and MDA-MB-436 (D and H) cells were transfected with miR-873 and control microRNA. Cells were counted in five random fields per well at 40× after 6 h for migration and after 24 h for invasion. The percentages of open area (A–D) and invading cells (E–H) in the miR-873 treatment group were calculated compared with the control group. Treatment with miR-873 significantly decreased the migration and invasion capacities of PDAC and TNBC cells compared with control cells. Data represent means + SE of three independent experiments. $^{*}p < 0.05$, $^{**}p < 0.01$, $^{***}p < 0.001$. (I) Expression levels of p-ERK (Thr202/Tyr204), ERK, p-AKT, and AKT were determined by western blot analysis in PDAC and TNBC cells in which miR-873 was ectopically expressed. GAPDH was used as a loading control.

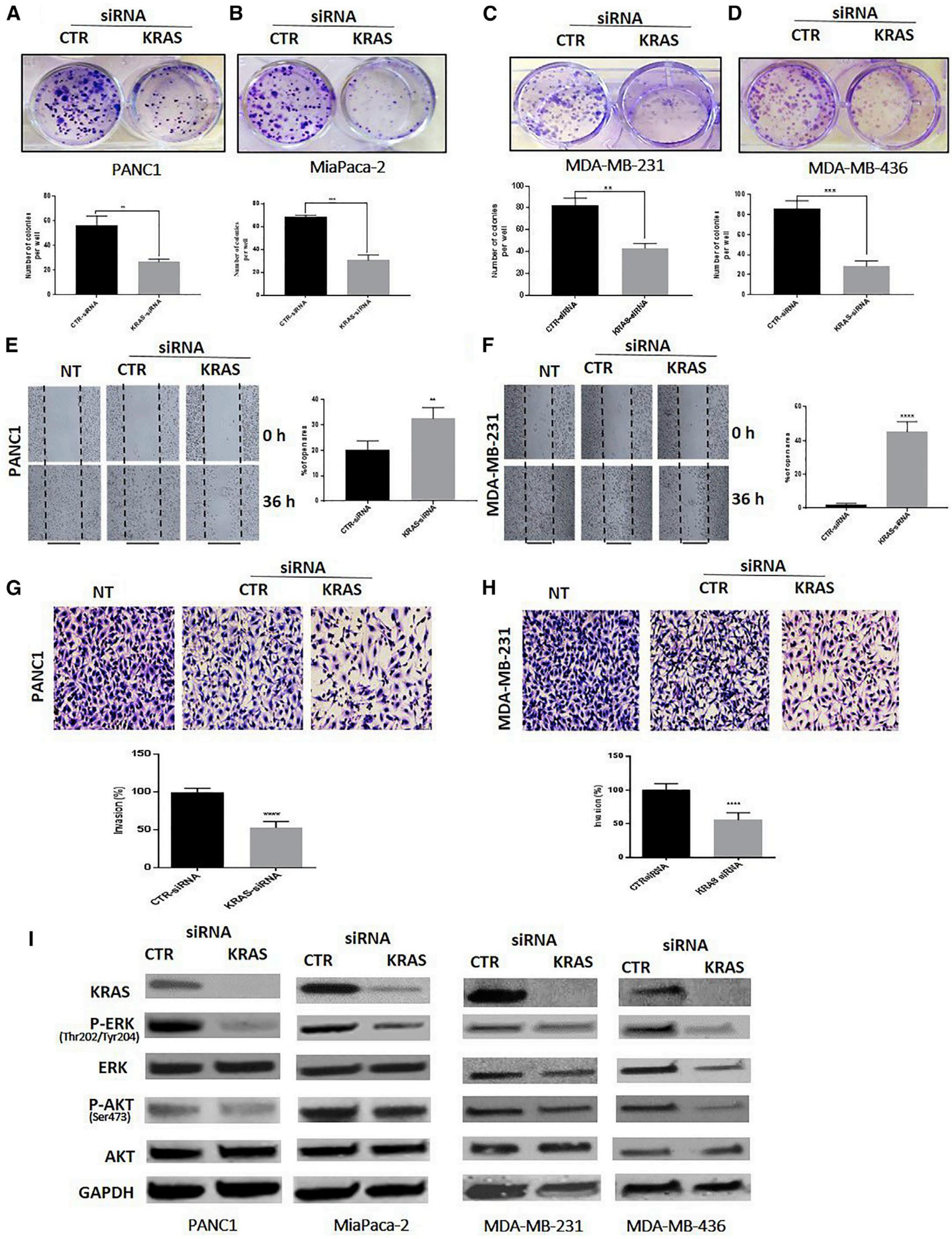

Figure 5. Silencing KRAS Recapitulates the Effects of miR-873 on Proliferation, Migration, and Invasion in PDAC and TNBC Cells

(A–H) The effect of silencing KRAS through transfection with KRAS siRNA or control siRNA on PANC1 (A), MiaPaCa-2 (B), MDA-MB-231 (C), and MDA-MB-436 (D) cells was determined by a colony-forming assay (A–D), migration assay (E and F), and invasion assay (G and H). Lower panels (for colony and invasion assays) and right panel (for migration assay) represent quantification of the number of colonies, migrating cells, and invading cells. Data represent means + SE of three independent experiments. **p < 0.01, ***p < 0.001, ****p < 0.0001. (I) Expression levels of KRAS, p-ERK (Thr202/Tyr204), ERK, p-AKT (Ser473), and AKT were determined by western blot analysis in PDAC (left panel) and TNBC (right panel) cells in which KRAS was silenced by siRNA. GAPDH was used as a loading control.

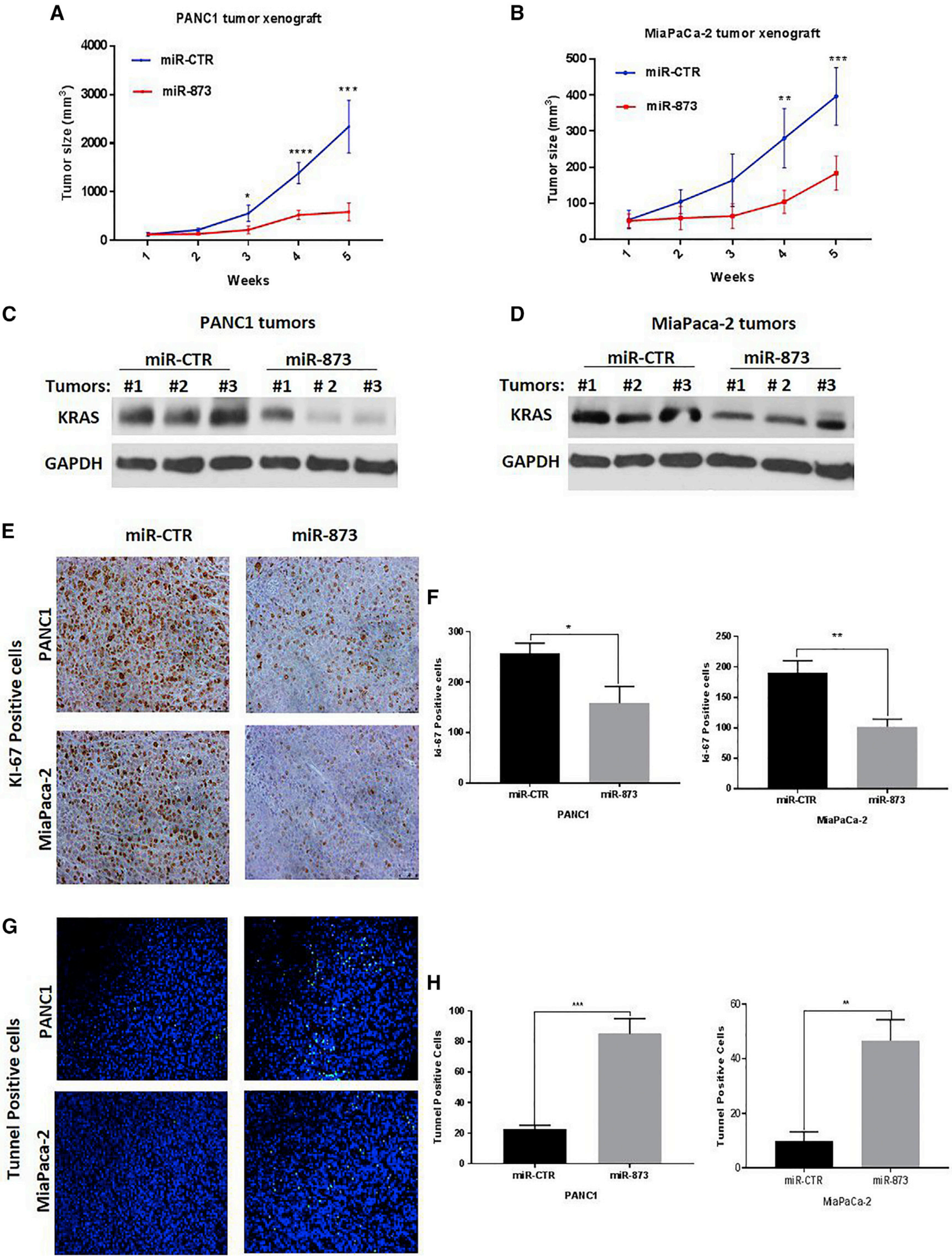

Figure 6. *In Vivo* Systemic Administration of Nanoparticle miR-873 Inhibits Tumor Growth and Decreases Tumoral Expression of KRAS in PDAC Tumor Xenografts

(A and B) PANC1 (A) and MiaPaCa-2 (B) tumor-bearing mice were treated with nanoparticles incorporating either control miRNA (miR-CTR) or miR-873 mimic (0.3 mg/kg [8 μg/mouse]) intravenously once per week for 5 weeks (five mice per group). Tumor volumes were measured weekly and are shown as means ± SE. *$p < 0.05$, **$p < 0.01$,

(legend continued on next page)

analyzed by western blot for the effects of miR-873 delivery on KRAS expression. Tumors from miR-873-treated mice showed reduced expression of KRAS compared with tumors from mice treated with control miRNA (Figures 6C, 6D, 7C, and 7D). Furthermore, there were no significant changes in mouse body weights at the end of the treatment period (Figures S10A and S10B), suggesting that miR-873-based delivery exerted no observed side effects.

We used immunohistochemistry staining for Ki-67 in isolated tumor tissues to examine the biological effects of miR-873 on tumor cell proliferation.[33] The number of Ki-67-positive tumor cells was significantly lower in tumors from mice treated with miR-873 than in those treated with control miRNA (PANC1, $p = 0.0123$; MiaPaCa-2, $p = 0.0029$; Figures 6E and 6F; MDA-MB-231, $p = 0.0178$; MDA-MB-436, $p = 0.006$; Figures 7E and 7F). Additionally, treatment with miR-873 significantly increased the number of TUNEL (terminal deoxynucleotidyl transferase-mediated dUTP nick end labeling)-positive cells compared with control miRNA (PANC1, $p = 0.005$; MiaPaCa-2, $p = 0.003$; Figures 6G and 6H; MDA-MB-231, $p = 0.0006$; MDA-MB-436, $p = 0.0028$; Figures 7G and 7H), suggesting that miR-873 has a pro-apoptotic effect *in vivo*. *In vivo* findings were consistent with the *in vitro* results observed in the cell proliferation assays, demonstrating that miR-873 has a tumor-suppressive function.

Overall, our results indicate that *in vivo* restoration of miR-873 expression inhibits tumor growth in PDAC and TNBC tumors through significant suppression of cell proliferation and induction of apoptosis.

Furthermore, our reverse-phase protein array (RPPA) analysis revealed that miR-873 transfection caused downregulation for many proteins related to the RAS/MAPK pathway, such as P-ERK, S6_pS240-pS244, and S6_pS235-pS236. By using ingenuity pathway analysis according to the RPPA results, miR-873 treatment led to significant changes in proteins and canonical pathways related to cancer signaling, such as cell cycle, angiogenesis, and colony formation (Figures 8A–8C; Figures S11A and S11B).

DISCUSSION

The findings presented here suggest that miR-873 is a clinically significant tumor suppressor miRNA whose reduced expression is associated with poor clinical prognosis and survival in patients with PDAC and TNBC. Restoration of miR-873 in PDAC and TNBC cells directly and significantly suppresses KRAS and its downstream ERK-MAPK- and PI3K-signaling pathways, which is one of the major drivers of PDAC and TNBC cell proliferation, invasion, and tumor growth. Our study also provides the first evidence that therapeutic replacement of miR-873 using systemically injected nanodelivery inhibits tumor growth in four different PDAC and TNBC models.

Activating mutations of KRAS are detected in more than 90% of patients with PDAC, and overexpression of KRAS and activation of the RAS/MAPK pathway are frequently observed in TNBC;[13,14] these major oncogenic activities represent potential molecular targets in these cancers.[17–19] Although molecular findings provide a strong rationale for developing novel therapeutic treatments targeting KRAS, direct inhibition of KRAS has proven to be very challenging,[20] leading KRAS to be classified as an undruggable target.[34] Therefore, there is a crucial demand to discover more effective treatments for pancreatic and breast cancers that harbor KRAS mutations.

In the current study, we found that miR-873 has a very low expression in PDAC and TNBC cells compared with normal cells, suggesting that miR-873 is frequently dysregulated in pancreatic and breast tumors. To date, several studies have described the role of miR-873 in different types of cancers, including breast cancer,[35] glioma,[36] lung adenocarcinoma,[37] ovarian cancer,[38] and colorectal cancer.[39] miR-873 was reported to inhibit the expression of genes such as *CDK3*,[35] *IGF2BP1*,[40] *Bcl-2*,[36] and *TRAF5* and *TAB1*.[39] However, the biological roles of miR-873 and its direct functional targets in PDAC and TNBC have not yet been elucidated. A key finding in our study was the regulation of KRAS by miR-873, which was shown to exert a tumor-suppressive effect in PDAC and TNBC, both *in vitro* and *in vivo*.

Oncogenic KRAS promotes pancreatic tumorigenesis through the activation of multiple downstream pathways, including RAF/MEK/ERK, MAPK, PI3K/Akt, Bad, and nuclear factor κB (NF-κB).[41,42] Inhibition of KRAS and its downstream pathways has been shown to reduce tumor growth and enhance gemcitabine chemotherapeutic efficacy against pancreatic cancer.[43,44] Downstream effectors of KRAS signaling in pancreatic cancer have been widely explored. Among them, Ras/Raf/ERK and Ras/PI3K/AKT signals are major effector pathways in PDAC tumorigenesis,[44] especially through cell cycle regulation.[45] Recent observation showed that ERK could function with AKT synergistically to promote PDAC tumorigenesis.[46] Furthermore, the expression of miR-873 in PDAC and TNBC cells inhibited the AKT and ERK pathways by directly targeting KRAS and inhibiting its translation. Thus, key effectors in KRAS signaling may be tightly regulated by miR-873 in the pancreas, and miR-873 restoration could effectively target Ras/Raf/ERK and Ras/PI3K/AKT to suppress PDAC and TNBC growth.

As our multiple *in vivo* studies suggested, targeting of KRAS by miR-873 is expected to be a highly efficient therapeutic strategy in PDAC, TNBC, and other KRAS-driven cancers. In breast cancer, KRAS/MAPK signaling plays an important role in growth signaling from the extracellular environment.[47,48] Activation of the KRAS/MAPK-signaling pathway induces numerous responses in cancer

***$p < 0.001$, ****$p < 0.0001$. (C–H) Nanoparticle delivery of miR-873 inhibits KRAS expression (C and D) and intratumoral proliferation (E and F) and induces apoptosis (G and H) in PDAC orthotopic xenograft mouse models. Immunohistochemical staining was used to evaluate the expression of proliferation marker Ki-67 and *in vivo* apoptosis marker TUNEL in PANC1 and MiaPaCa-2 mouse xenografts treated with nanoparticle miR-873 or control miRNA mimic (E and G). Positively stained cells in both treatment groups were quantified (F and H). Data represent means + SE. *$p < 0.05$, **$p < 0.01$, ***$p < 0.001$.

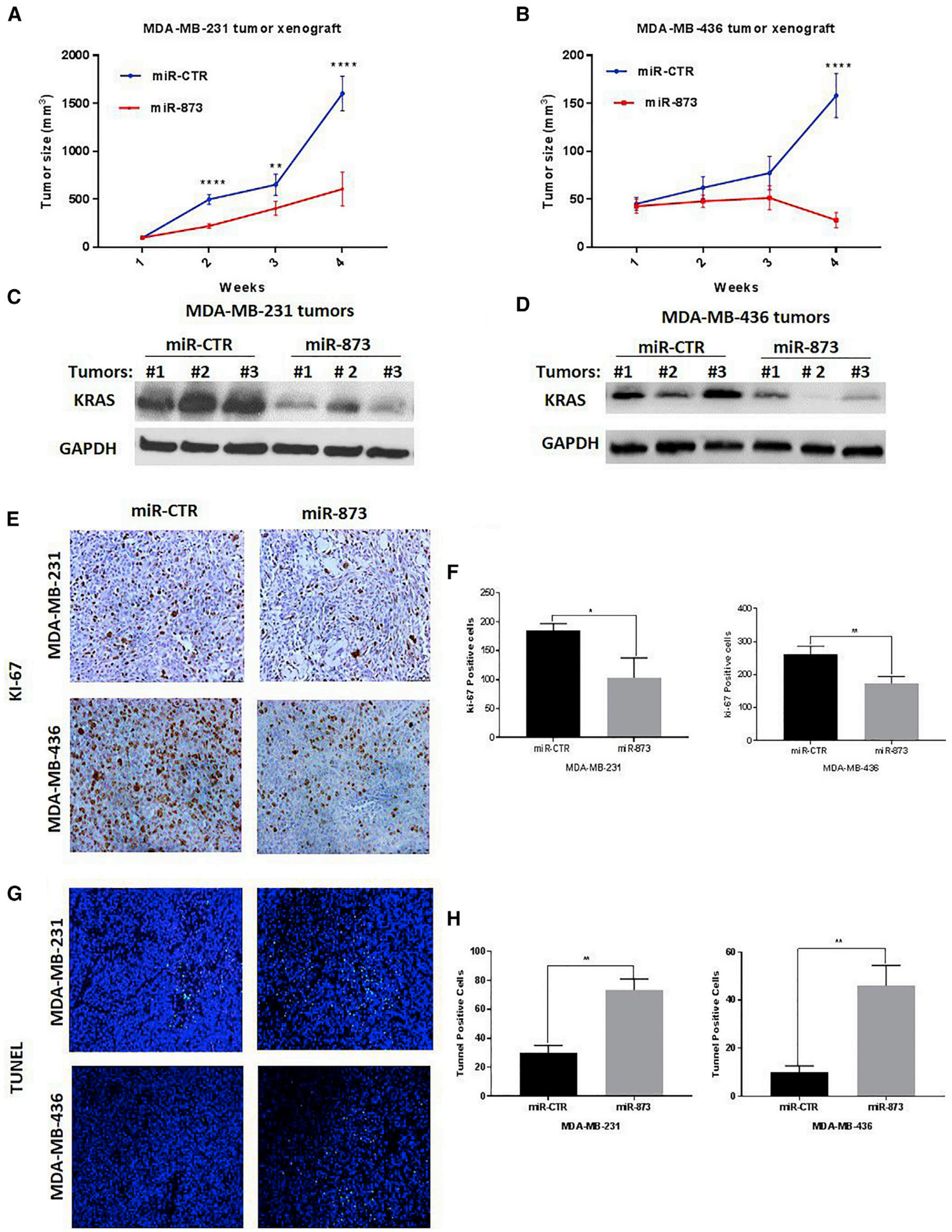

Figure 7. *In Vivo* Systemic Injection of Nanoparticle miR-873 Inhibits Tumor Growth and Decreases Tumoral Expression of KRAS in Orthotopic Xenograft TNBC Models

(A and B) MDA-MB-231 (A) and MDA-MB-436 (B) tumor-bearing mice were treated with nanoparticles incorporating either control miRNA (miR-CTR) or miR-873 mimic (0.3 mg/kg [8 μg/mouse]) intravenously once per week for 4 weeks (five mice per group). Tumor volumes were measured weekly and are shown as means ± SE. **$p < 0.01$,

(legend continued on next page)

cells, regulating cell proliferation, differentiation, migration, and invasion. In addition, noncanonical activation of KRAS/MAPK signaling has been shown to be regulated in breast cancer tissues by increased expression levels of EGFR, HER2, and insulin-like growth factor receptor.[49–51] Furthermore, germline polymorphism in the KRAS 3′ UTR is considered a genetic marker of increased risk of TNBC progression in premenopausal women.[52]

Previous studies showed that KRAS was regulated by several miRNAs, including let-7, miR-16, miR-143, and miR-1298 in breast cancer cells; miR-4689 in colon cancer; and miR-193b in PDAC cells.[53–56] In the current study, we demonstrated that miR-873 is a clinically significant miRNA and reduced expression of miR-873 is associated with significantly reduced overall survival, whereas restoration of miR-873 expression decreases cell proliferation, migration and invasion, and tumorigenesis by downregulating KRAS expression levels in PDAC and TNBC tumor models. Meanwhile, these effects were rescued by the overexpression of KRAS. Therefore, miR-873 may be one of the most important miRNA regulators of the KRAS oncogene, and these findings have the potential for translation into clinical studies for therapeutic applications. Interestingly, miR-873 was found to significantly induce apoptosis in PDAC and TNBC cells. This observation was confirmed by different methods (western blot detection of the apoptosis markers PARP, caspase-3, and caspase-7 and flow cytometry analysis of the Annexin V staining). This result is in parallel with that of the previous finding of Chen et al.,[36] who showed that this miRNA per se induced apoptosis in glioma. Most importantly, the inhibition of tumor growth in an orthotopic xenograft mouse model of PDAC and TNBC via lipid-based nanoparticle delivery of miR-873 suggests that strategies targeting the miR-873/KRAS axis may provide broad antitumor effects through the inhibition of multiple oncogenic pathways.

In summary, our findings provide new insight into the posttranscriptional regulation of mutant and wild-type KRAS by miR-873 in PDAC and TNBC tumorigenesis. In particular, we showed that miR-873 regulates important hallmarks of cancer, including cell proliferation, apoptosis, and invasion, through the targeting of multiple oncogenic routes involving KRAS-induced ERK/AKT signaling. Hence, these findings may have important translational implications; miR-873-based therapy might be an attractive therapeutic strategy to treat PDAC and TNBC.

MATERIALS AND METHODS

Survival Analyses

To investigate the relationship between KRAS or miR-873 expression and overall survival, clinical data associated with 172 basal-like breast tumors[57,58] and 177 pancreatic tumors[59] were obtained from TCGA cohorts. Patients were divided into KRAS or miR-873 high or low groups based on median KRAS or miR-873 expression for each tumor type. For each analysis, differences in overall survival (OS) were calculated by a log-rank test, and the hazard ratio (HR) is reported.

To investigate the differences in KRAS mRNA expression in tumor versus normal tissue, gene expression data from 14 basal-like breast cancer samples and 45 pancreatic samples with matched tumor and adjacent normal tissue were obtained from TCGA and GEO: GSE28735, respectively.[60] Differences in KRAS expression for basal-like breast cancer and pancreatic cancer were calculated using a Student's t test, with $p < 0.05$ considered statistically significant

Cell Lines and Cell Culture Conditions

The human mammary epithelial cell line MCF10A; breast cancer cell lines MDA-MB-436, MDA-MB-231, MDA-MB-453, BT-20, HCC1937, SKBR3, T47D, and HEK293; normal HPDE cells; and human PDAC cell lines PANC1, BxPC-3, MiaPaCa-2, and Capan-2 were purchased from the American Type Culture Collection (Manassas, VA). All breast cancer cells and PANC1 and MiaPaCa-2 cells were cultured in DMEM/F12 (Sigma, St. Louis, MO). BxPC-3 and Capan-2 cells were cultured in RPMI-1640 medium supplemented with 10% fetal bovine serum (FBS) and a 100-U/mL penicillin-streptomycin solution (Sigma). All media were supplemented with 10% FBS and a 100-U/mL penicillin-streptomycin solution. MCF-10A cells were maintained in a nutrient mixture consisting of DMEM/F12 supplemented with 5% horse serum, epidermal growth factor, hydrocortisone, insulin, and cholera toxin. All cultured cells were incubated at 37°C in a water-saturated 95% air-5% CO_2 atmosphere.

Transfections with miRNA Mimics and siRNAs

At 24 h after seeding of the cells, we transfected them with 100 nM mimics of miR-873-5p, 100 nM control miRNA (Ambion, Austin, TX), 50 nM KRAS, and/or control siRNA (Sigma) by using HiPerFect transfection reagent (QIAGEN, Germantown, MD) in Opti-MEM negative Serum Medium (Life Technologies, Carlsbad, CA), according to the manufacturer's protocol (mature miR-873-5p sequence: 5′-GCAGGAACUUGUGAGUCUCCU-3′). At 6 h after transfection, cells were kept in a culture medium containing 10% FBS for up to 72 h.

Cell Viability and Colony Formation Assays

The viability of TNBC and PDAC cells was analyzed using the MTS assay as previously described.[61] A total of 1×10^3 to 2×10^3 cells/well were seeded in 96-well plates. Cells were incubated overnight and then treated with a synthetic RNA oligonucleotide. Cell viability was determined at 24, 48, 72, and 96 h using 5 mg/mL MTS. We analyzed the plates at a wavelength of 490 nm in a VMax kinetic

***$p < 0.001$, ****$p < 0.0001$. (C–H) Nanoparticle delivery of miR-873 inhibits KRAS expression (C and D) and intratumoral proliferation (E and F) and induces apoptosis (G and H) in TNBC orthotopic xenograft mouse models. Immunohistochemical staining was used to evaluate the expression of proliferation marker Ki-67 and *in vivo* apoptosis marker TUNEL in MDA-MB-231 and MDA-MB-436 mouse xenografts treated with nanoparticle miR-873 or control miRNA mimic (E and G). Positively stained cells in both treatment groups were quantified (F and H). Data represent means + SE. *$p < 0.05$, **$p < 0.01$.

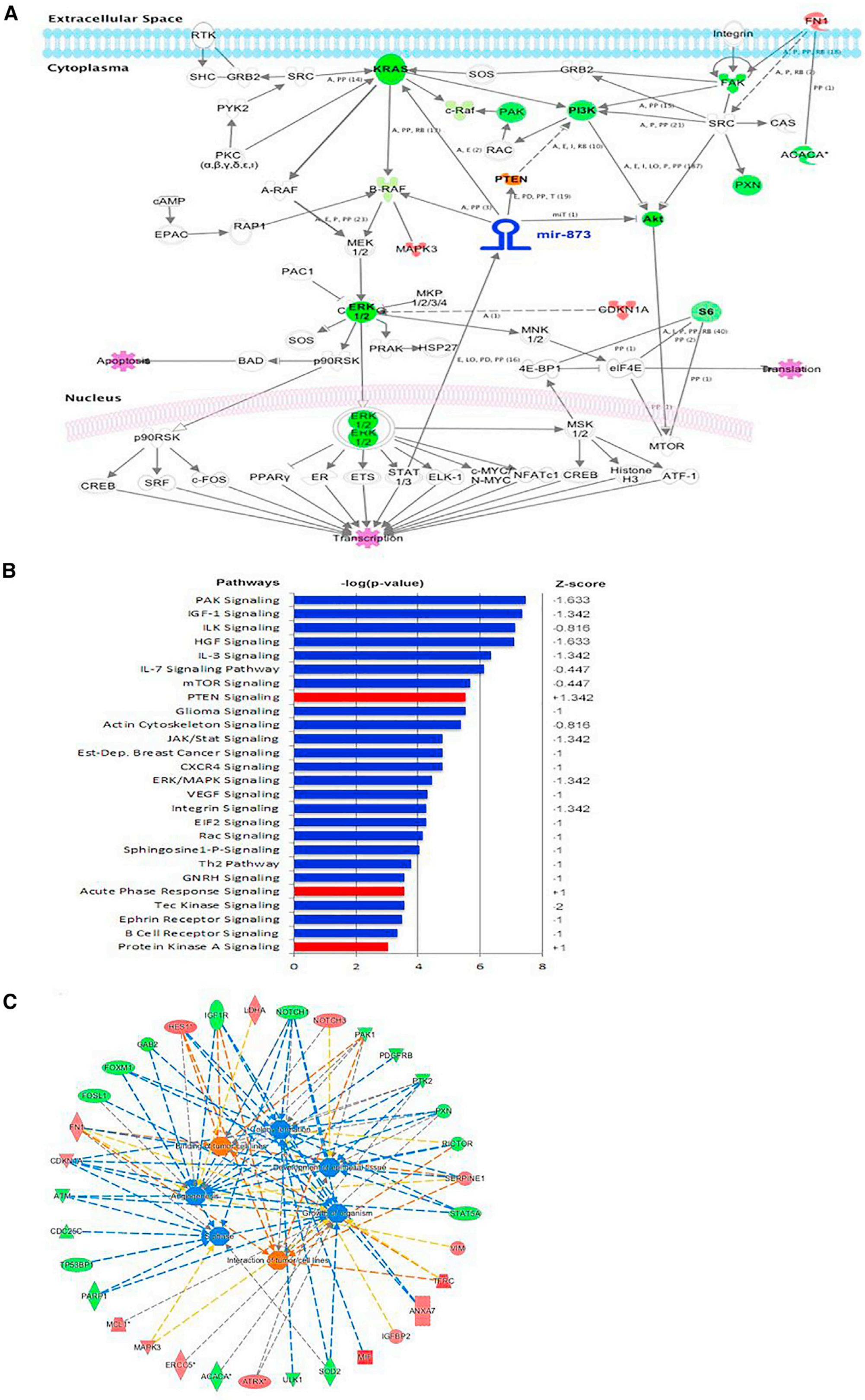

(legend on next page)

ELISA microplate reader (Molecular Devices, Sunnyvale, CA). For the colony formation assay, cells were seeded in 12-well plates at a low density (500 cells/plate), transfected with miRNA or siRNA and the respective negative controls, and allowed to grow until visible colonies appeared. The colonies were then stained with crystal violet and counted. Each experiment was performed in triplicate.

RNA Isolation and Real-Time qPCR

MiRNeasy Mini Kit (QIAGEN) was used to isolate total RNA according to the manufacturer's guidelines, and a total of 1,000 ng RNA was used as a template and then reverse transcribed to cDNA using the qScript miRNA cDNA Synthesis Kit (Quanta BioSciences, Beverly, MA). The expression level of miR-873 was measured with the PerfeCTa miRNA Assay Kit (Quanta BioSciences) using miRNA primers from Quanta BioSciences, using real-time qPCR and normalized to the level of U6 small nuclear RNA (RNU6; Quanta BioSciences), which was used as an endogenous control.

To quantify KRAS mRNA, we synthesized cDNA from the isolated RNA using a QuantiTect reverse transcription kit (QIAGEN). qRT-PCR was carried out with BX-384 Bio-Rad using the QuantiTect SYBR Green PCR kit (QIAGEN), according to the manufacturer's protocol. All reactions were performed in triplicate. KRAS mRNA expression levels were normalized to the internal control, glyceraldehyde 3-phosphate dehydrogenase (GAPDH). The sequences of the sense and anti-sense KRAS primers were 5′-ATTGT-GAATGTTGGTGT-3′ and 5′-GAAGGTCTCAACTGAAATT-3′, respectively. The sequences of the sense and anti-sense GAPDH primers were 5′-CAAGGTCATCCATGA CAACTTTG-3′ and 5′-GTCCACCACCCTGTTGCTGTAG-3′, respectively. Relative quantification of mRNA and miRNA expression was calculated using the comparative threshold cycle ($2^{-\Delta\Delta Ct}$) method.

Protein Extraction and Western Blot Analysis

At 48 h after miRNA transfection, cells were lysed in 1× radioimmunoprecipitation assay (RIPA) buffer (Thermo Scientific, Waltham, MA) containing protease and phosphatase inhibitors. Lysates were centrifuged, supernatants were collected, and the total protein concentration in lysates was assessed using the Pierce BCA protein assay kit (Thermo Scientific). Next, lysates were resuspended in Laemmli loading buffer (Bio-Rad, Hercules, CA) and heated at 95°C for 5 min. Western blot analysis was performed as previously reported.[62] The expression of proteins was detected using specific antibodies for KRAS (Santa Cruz Biotechnology, Santa Cruz, CA), p-ERK1/2$^{(Thr202/Tyr204)}$, ERK 1/2, p-AKT$^{(Ser473)}$, AKT, p-C-RAF$^{(Ser338)}$, C-RAF, p-MEK1/2$^{(Ser217/221)}$, MEK, caspase-3, cleaved caspase-3, PARP, cleaved-PARP caspase-7, cleaved caspase-7, GAPDH (Cell Signaling Technology, Danvers, MA), and β-actin (Sigma) and the corresponding horseradish peroxidase-conjugated secondary antibodies (Cell Signaling Technology).

Luciferase Reporter Assay for miR-873 Target Gene Binding and Expression

PANC1, MDA-MB-231, and HEK293 normal epithelial cells were transfected with pEZX-MT06 miRNA reporter vectors containing the binding sites for miR-873 in the 3′ UTR of *KRAS* and the luciferase gene (GeneCopoeia, Rockville, MD). Cells were plated (5 × 10^4 cells per well) in each well of a 24-well plate 24 h before transfection. The cells were transfected with the pEZX-MT06 vector (200 ng) and 50 nM miR-873 mimic or control miRNA. Luciferase activity was measured 48 h after transfection using the Luc-Pair miR Luciferase Assay (GeneCopoeia). For each sample, firefly luciferase activity was normalized to *Renilla* luciferase activity.

RPPA

RPPA analysis was performed at the Functional Proteomics RPPA Core Facility of The University of Texas MD Anderson Cancer Center. The RPPA assay was performed as previously described.[61,63]

Apoptosis

Cell apoptosis was assessed by Annexin V/PI staining using a fluorescein isothiocyanate (FITC) apoptosis detection kit (BD Biosciences), according to the manufacturer's protocol. Apoptotic cells were analyzed with a FACSCalibur flow cytometer (BD Biosciences). We used CellQuest Pro software (BD Biosciences) to detect the number of apoptotic cells. Apoptosis was also assessed by detection of the cleavage of caspase-3, caspase-7, and PARP via western blotting.

Wound-Healing Assay

To measure cell motility and migration, we used an *in vitro* wound-healing assay. PANC1, MiaPaCa-2, and BxPC-3 cells (4 × 10^5 cells/well), as well as MDA-MB-231 and MDA-MB-436 cells (2 × 10^5 cells/well), were plated in six-well plates and cultured in medium containing 10% FBS. After 24 h of incubation, cells were transfected with control miRNA or miR-873 mimic, KRAS siRNA, or control siRNA. The second day after transfection, we used a 200-μL sterile pipette tip to do straight scratch on the confluent cell layers. Then cells were photographed using a phase-contrast microscope (Nikon Eclipse TE-200-U) at 0 h. The cells were continued in incubation, and images were taken again at 24 and 36 h. The wound healing was displayed by comparing photographs taken at 0 h with those taken 24 and 36 h later. At least 5 random non-overlapping pictures for each

Figure 8. Schematic Model of the Regulatory Pathway Involving miR-873

Ectopic overexpression of miR-873 inhibits multiple downstream signaling pathways. (A) Ingenuity pathway analysis of the canonical pathways and proteins that were significantly altered by ectopic expression of miR-873 in cells after reverse-phase protein array (RPPA) analysis. Red indicates that the expression level of that protein was higher in miR-873-transfected cells than in control miRNA-transfected cells, and green indicates that the expression level was lower. (B and C) Graphs produced by RPPA analysis of PANC1 cells treated with miR-873 or control mimic for 72 h show downregulation of potential target pathways. Canonical pathway analysis showed that multiple interconnected pathways (inhibited [blue] and activated [red]) related to cancer signaling were altered upon miR-873 transfection ($p < 0.01$).

experiment were tested and quantitated using ImageJ software (NIH, Bethesda, MD).

Matrigel Invasion Assay

Transwell inserts (4 μm pore size; Fisher Scientific) coated with a Matrigel basement membrane (0.7 mg/mL; BD Biosciences) were used for this assay. After 48-h transfection with 100 nM miR-873, control miRNA, KRAS siRNA, or control siRNA, 8×10^4 (PANC1, MDA-MB-231, and MDA-MB-436) and 100×10^4 (MiaPaCa-2) cells were resuspended in 500 μL serum-free medium and then added to the upper chamber of the insert. Lower chambers were supplied with 500 μL medium supplemented with 10% FBS. After 24 h of incubation, non-invading cells on the upper chamber of the filter were cleared with cotton swabs. Cells that invaded through the Matrigel onto the lower chamber were fixed, stained with the Hema-3 Stain System (Fisher Scientific), and photographed. All experiments were performed in triplicate and invaded cells to the lower side of the filter were counted in at least 5 fields and expressed as a percentage of invasion.

Establishment of Transient KRAS-Overexpressing Cells

PANC1 and MDA-MB-231 cells were transfected with plasmids containing the specified mutated open reading frame (ORF) clones (no 3′ UTR) of KRAS. The KRAS G12D mutant construct (pCMV6-Entry-KRAS G12D, RC400104), KRAS G13D mutant construct (pCMV6-Entry-KRAS G12D, RC400116), and control vector (pCMV6-AC-GFP, PS100010) were purchased from OriGene (Rockville, MD, USA). KRAS protein expression was verified by western blotting.

Tumor Xenograft Models

All studies were conducted according to an experimental protocol approved by the MD Anderson Institutional Animal Care and Use Committee. To generate orthotopic TNBC tumors, we obtained athymic female nude mice (4–5 weeks old) from the Department of Experimental Radiation Oncology at MD Anderson, and we orthotopically injected TNBC cells (MDA-MB-231, 2×10^6 in 20% matrigel; or MDA-MB-436, 3×10^6 in 20% matrigel) into the mammary fat pad of each mouse. For PDAC tumor models, we established tumors by injecting PANC1 (5×10^6 in 30% matrigel) or MiaPaCa-2 cells (2×10^6 in 20% matrigel) into the right flank of each mouse. At 14 days after injection, when the size of the tumor reached about 3–5 mm, we started liposomal-miRNA treatment as previously described.[64] Each mouse was injected with miR-873 or control miRNA (0.3 mg/kg equivalent of 8 μg/mouse once a week) in a volume of 100 μL for 5 weeks (total of five intravenous [i.v.] injections) through the tail vein. Tumor sizes were measured regularly with an electronic caliper. When treatment was completed, mice were euthanized with CO_2 and weighed to measure tumor growth, and tumor tissues were reserved for further analysis.

Immunohistochemical Analysis

Immunostaining for Ki-67 was processed to estimate cell proliferation, in formalin-fixed paraffin-embedded tumor tissues, according to the manufacturer's protocol. Briefly, slides were deparaffinized by heating at 55°C for 30 min, then exposed to xylene, and rehydrated via a series of descending series of ethyl alcohol solution. After that, we incubated slides in antigen retrieval solution (Dako) at 95°C for 30 min. Endogenous peroxidases activity was blocked by putting slides in methanol containing 3% hydrogen peroxide for 15 min. The slides were incubated with Ki-67 primary antibody (Abcam, Cambridge, MA) at 4°C overnight. Then, secondary antibodies were applied to the sections at room temperature for 1 h. Afterward, the sections were counterstained with hematoxylin for 30 s and analyzed by microscopy (Nikon Eclipse TE-200-U).

TUNEL Assay

The TUNEL assay (Promega, Madison, WI) was used to detect and quantify *in vivo* apoptotic cells in tumor tissues by measuring nuclear DNA fragmentation, according to the manufacturer's recommended protocol. The assay was performed as previously described.[61]

Statistical Analysis

Unless otherwise specified, data were expressed as the mean ± SE of three independent experiments. The Student's t test was to determine statistical significance (set at $p < 0.05$). Student's t tests and ANOVA were calculated using GraphPad software.

SUPPLEMENTAL INFORMATION

Supplemental Information includes eleven figures and one table and can be found with this article online at https://doi.org/10.1016/j.omtn.2018.11.019.

AUTHOR CONTRIBUTIONS

H.A.M. and B.O. conceived and coordinated the study and wrote the paper. R.B., H.A.M., N.N.K., and E.P.Z. helped with some *in vitro* assays. A.C., J.S., and S.W. analyzed RPPA, generated the heatmap, and did ingenuity pathway analyses (IPAs). P.K., K.K., and M.L.G. analyzed overall survival of the study. H.A.M., N.N.K., and N.K. performed immunohistochemical (IHC) analysis for *in vivo* tissue samples and contributed to figure preparation. N.N.K. contributed to manuscript writing and figure preparation. H.A.M. and N.N.K. contributed to overexpression of the gene. G.L.-B., G.A.C., A.A.H.A.A., T.M.A., and A.A.A. contributed to writing the manuscript. B.O., C.R.-A., and N.N.K. prepared nanoliposomal particles incorporating miRs and performed *in vivo* studies and the animal studies. H.A.M. and N.N.K. provided technical assistance for this study. All authors analyzed the results and approved the final version of the manuscript.

CONFLICTS OF INTEREST

The authors declare no competing interests.

ACKNOWLEDGMENTS

This study was supported by the funding from The University of Texas MD Anderson Cancer Center Sequencing and Non-coding RNA Program, NIH/National Cancer Institute grant U54 to MD Anderson, and the Ministry of High Education, Cultural Affairs, and

Mission Sector, Joint-Supervision Program, Egypt. The Functional Proteomics RPPA Core Facility at MD Anderson was used as part of this research.

REFERENCES

1. Pylayeva-Gupta, Y., Grabocka, E., and Bar-Sagi, D. (2011). RAS oncogenes: weaving a tumorigenic web. Nat. Rev. Cancer *11*, 761–774.
2. Tao, S., Wang, S., Moghaddam, S.J., Ooi, A., Chapman, E., Wong, P.K., and Zhang, D.D. (2014). Oncogenic KRAS confers chemoresistance by upregulating NRF2. Cancer Res. *74*, 7430–7441.
3. Zuber, J., Tchernitsa, O.I., Hinzmann, B., Schmitz, A.C., Grips, M., Hellriegel, M., Sers, C., Rosenthal, A., and Schäfer, R. (2000). A genome-wide survey of RAS transformation targets. Nat. Genet. *24*, 144–152.
4. Crespo, P., and León, J. (2000). Ras proteins in the control of the cell cycle and cell differentiation. Cell. Mol. Life Sci. *57*, 1613–1636.
5. Wu, Y., Zhuang, Y., Han, M., Xu, T., and Deng, K. (2009). Ras promotes cell survival by antagonizing both JNK and Hid signals in the Drosophila eye. BMC Dev. Biol. *9*, 53.
6. Tuveson, D.A., Shaw, A.T., Willis, N.A., Silver, D.P., Jackson, E.L., Chang, S., Mercer, K.L., Grochow, R., Hock, H., Crowley, D., et al. (2004). Endogenous oncogenic K-ras(G12D) stimulates proliferation and widespread neoplastic and developmental defects. Cancer Cell *5*, 375–387.
7. Bos, J.L. (1989). ras oncogenes in human cancer: a review. Cancer Res. *49*, 4682–4689.
8. Bos, J.L., Fearon, E.R., Hamilton, S.R., Verlaan-de Vries, M., van Boom, J.H., van der Eb, A.J., and Vogelstein, B. (1987). Prevalence of ras gene mutations in human colorectal cancers. Nature *327*, 293–297.
9. Downward, J. (2003). Role of receptor tyrosine kinases in G-protein-coupled receptor regulation of Ras: transactivation or parallel pathways? Biochem. J. *376*, e9–e10.
10. Schubbert, S., Shannon, K., and Bollag, G. (2007). Hyperactive Ras in developmental disorders and cancer. Nat. Rev. Cancer *7*, 295–308.
11. Hollestelle, A., Elstrodt, F., Nagel, J.H., Kallemeijn, W.W., and Schutte, M. (2007). Phosphatidylinositol-3-OH kinase or RAS pathway mutations in human breast cancer cell lines. Mol. Cancer Res. *5*, 195–201.
12. Bamford, S., Dawson, E., Forbes, S., Clements, J., Pettett, R., Dogan, A., Flanagan, A., Teague, J., Futreal, P.A., Stratton, M.R., and Wooster, R. (2004). The COSMIC (Catalogue of Somatic Mutations in Cancer) database and website. Br. J. Cancer *91*, 355–358.
13. Kopp, F., Wagner, E., and Roidl, A. (2014). The proto-oncogene KRAS is targeted by miR-200c. Oncotarget *5*, 185–195.
14. Giltnane, J.M., and Balko, J.M. (2014). Rationale for targeting the Ras/MAPK pathway in triple-negative breast cancer. Discov. Med. *17*, 275–283.
15. She, Q.B., Chandarlapaty, S., Ye, Q., Lobo, J., Haskell, K.M., Leander, K.R., DeFeo-Jones, D., Huber, H.E., and Rosen, N. (2008). Breast tumor cells with PI3K mutation or HER2 amplification are selectively addicted to Akt signaling. PLoS ONE *3*, e3065.
16. Jancík, S., Drábek, J., Radzioch, D., and Hajdúch, M. (2010). Clinical relevance of KRAS in human cancers. J. Biomed. Biotechnol. *2010*, 150960.
17. Pratilas, C.A., Taylor, B.S., Ye, Q., Viale, A., Sander, C., Solit, D.B., and Rosen, N. (2009). (V600E)BRAF is associated with disabled feedback inhibition of RAF-MEK signaling and elevated transcriptional output of the pathway. Proc. Natl. Acad. Sci. USA *106*, 4519–4524.
18. Balko, J.M., Mayer, I.A., Sanders, M.E., Miller, T.W., Kuba, M.G., Meszoely, I.M., Wagle, N., Garraway, L.A., and Arteaga, C.L. (2012). Discordant cellular response to presurgical letrozole in bilateral synchronous ER+ breast cancers with a KRAS mutation or FGFR1 gene amplification. Mol. Cancer Ther. *11*, 2301–2305.
19. Hoeflich, K.P., O'Brien, C., Boyd, Z., Cavet, G., Guerrero, S., Jung, K., Januario, T., Savage, H., Punnoose, E., Truong, T., et al. (2009). In vivo antitumor activity of MEK and phosphatidylinositol 3-kinase inhibitors in basal-like breast cancer models. Clin. Cancer Res. *15*, 4649–4664.
20. Gysin, S., Salt, M., Young, A., and McCormick, F. (2011). Therapeutic strategies for targeting ras proteins. Genes Cancer *2*, 359–372.
21. Pasquinelli, A.E. (2012). MicroRNAs and their targets: recognition, regulation and an emerging reciprocal relationship. Nat. Rev. Genet. *13*, 271–282.
22. Akbari Moqadam, F., Pieters, R., and den Boer, M.L. (2013). The hunting of targets: challenge in miRNA research. Leukemia *27*, 16–23.
23. Nelson, K.M., and Weiss, G.J. (2008). MicroRNAs and cancer: past, present, and potential future. Mol. Cancer Ther. *7*, 3655–3660.
24. Goto, Y., Kurozumi, A., Enokida, H., Ichikawa, T., and Seki, N. (2015). Functional significance of aberrantly expressed microRNAs in prostate cancer. Int. J. Urol. *22*, 242–252.
25. Esquela-Kerscher, A., and Slack, F.J. (2006). Oncomirs - microRNAs with a role in cancer. Nat. Rev. Cancer *6*, 259–269.
26. Goswami, C.P., and Nakshatri, H. (2014). PROGgeneV2: enhancements on the existing database. BMC Cancer *14*, 970.
27. Lewis, B.P., Shih, I.H., Jones-Rhoades, M.W., Bartel, D.P., and Burge, C.B. (2003). Prediction of mammalian microRNA targets. Cell *115*, 787–798.
28. Maragkakis, M., Reczko, M., Simossis, V.A., Alexiou, P., Papadopoulos, G.L., Dalamagas, T., Giannopoulos, G., Goumas, G., Koukis, E., Kourtis, K., et al. (2009). DIANA-microT web server: elucidating microRNA functions through target prediction. Nucleic Acids Res. *37*, W273–W276.
29. Betel, D., Wilson, M., Gabow, A., Marks, D.S., and Sander, C. (2008). The microRNA.org resource: targets and expression. Nucleic Acids Res. *36*, D149–D153.
30. Dweep, H., and Gretz, N. (2015). miRWalk2.0: a comprehensive atlas of microRNA-target interactions. Nat. Methods *12*, 697.
31. Hubbard, P.A., Moody, C.L., and Murali, R. (2014). Allosteric modulation of Ras and the PI3K/AKT/mTOR pathway: emerging therapeutic opportunities. Front. Physiol. *5*, 478.
32. Calvo, F., Agudo-Ibáñez, L., and Crespo, P. (2010). The Ras-ERK pathway: understanding site-specific signaling provides hope of new anti-tumor therapies. BioEssays *32*, 412–421.
33. Scholzen, T., and Gerdes, J. (2000). The Ki-67 protein: from the known and the unknown. J. Cell. Physiol. *182*, 311–322.
34. Zeitouni, D., Pylayeva-Gupta, Y., Der, C.J., and Bryant, K.L. (2016). KRAS Mutant Pancreatic Cancer: No Lone Path to an Effective Treatment. Cancers (Basel) *8*, E45.
35. Cui, J., Yang, Y., Li, H., Leng, Y., Qian, K., Huang, Q., Zhang, C., Lu, Z., Chen, J., Sun, T., et al. (2015). MiR-873 regulates ERα transcriptional activity and tamoxifen resistance via targeting CDK3 in breast cancer cells. Oncogene *34*, 3895–3907.
36. Chen, X., Zhang, Y., Shi, Y., Lian, H., Tu, H., Han, S., Peng, B., Liu, W., and He, X. (2015). MiR-873 acts as a novel sensitizer of glioma cells to cisplatin by targeting Bcl-2. Int. J. Oncol. *47*, 1603–1611.
37. Gao, Y., Xue, Q., Wang, D., Du, M., Zhang, Y., and Gao, S. (2015). miR-873 induces lung adenocarcinoma cell proliferation and migration by targeting SRCIN1. Am. J. Transl. Res. *7*, 2519–2526.
38. Wu, D.D., Li, X.S., Meng, X.N., Yan, J., and Zong, Z.H. (2016). MicroRNA-873 mediates multidrug resistance in ovarian cancer cells by targeting ABCB1. Tumour Biol. *37*, 10499–10506.
39. Gong, H., Fang, L., Li, Y., Du, J., Zhou, B., Wang, X., Zhou, H., Gao, L., Wang, K., and Zhang, J. (2018). miR-873 inhibits colorectal cancer cell proliferation by targeting TRAF5 and TAB1. Oncol. Rep. *39*, 1090–1098.
40. Wang, R.J., Li, J.W., Bao, B.H., Wu, H.C., Du, Z.H., Su, J.L., Zhang, M.H., and Liang, H.Q. (2015). MicroRNA-873 (miRNA-873) inhibits glioblastoma tumorigenesis and metastasis by suppressing the expression of IGF2BP1. J. Biol. Chem. *290*, 8938–8948.
41. Campbell, P.M., Groehler, A.L., Lee, K.M., Ouellette, M.M., Khazak, V., and Der, C.J. (2007). K-Ras promotes growth transformation and invasion of immortalized human pancreatic cells by Raf and phosphatidylinositol 3-kinase signaling. Cancer Res. *67*, 2098–2106.
42. Zhang, W., and Liu, H.T. (2002). MAPK signal pathways in the regulation of cell proliferation in mammalian cells. Cell Res. *12*, 9–18.
43. Russo, A., Bazan, V., Agnese, V., Rodolico, V., and Gebbia, N. (2005). Prognostic and predictive factors in colorectal cancer: Kirsten Ras in CRC (RASCAL) and TP53CRC collaborative studies. Ann. Oncol. *16* (*Suppl 4*), iv44–iv49.

44. Eser, S., Schnieke, A., Schneider, G., and Saur, D. (2014). Oncogenic KRAS signalling in pancreatic cancer. Br. J. Cancer *111*, 817–822.

45. Coleman, M.L., Marshall, C.J., and Olson, M.F. (2004). RAS and RHO GTPases in G1-phase cell-cycle regulation. Nat. Rev. Mol. Cell Biol. *5*, 355–366.

46. Collisson, E.A., Trejo, C.L., Silva, J.M., Gu, S., Korkola, J.E., Heiser, L.M., Charles, R.P., Rabinovich, B.A., Hann, B., Dankort, D., et al. (2012). A central role for RAF→MEK→ERK signaling in the genesis of pancreatic ductal adenocarcinoma. Cancer Discov. *2*, 685–693.

47. Haagenson, K.K., and Wu, G.S. (2010). The role of MAP kinases and MAP kinase phosphatase-1 in resistance to breast cancer treatment. Cancer Metastasis Rev. *29*, 143–149.

48. Dunn, K.L., Espino, P.S., Drobic, B., He, S., and Davie, J.R. (2005). The Ras-MAPK signal transduction pathway, cancer and chromatin remodeling. Biochem. Cell Biol. *83*, 1–14.

49. Salh, B., Marotta, A., Matthewson, C., Ahluwalia, M., Flint, J., Owen, D., and Pelech, S. (1999). Investigation of the Mek-MAP kinase-Rsk pathway in human breast cancer. Anticancer Res. *19* (1B), 731–740.

50. Eckert, L.B., Repasky, G.A., Ulkü, A.S., McFall, A., Zhou, H., Sartor, C.I., and Der, C.J. (2004). Involvement of Ras activation in human breast cancer cell signaling, invasion, and anoikis. Cancer Res. *64*, 4585–4592.

51. Lo, H.W., Hsu, S.C., and Hung, M.C. (2006). EGFR signaling pathway in breast cancers: from traditional signal transduction to direct nuclear translocalization. Breast Cancer Res. Treat. *95*, 211–218.

52. Paranjape, T., Heneghan, H., Lindner, R., Keane, F.K., Hoffman, A., Hollestelle, A., Dorairaj, J., Geyda, K., Pelletier, C., Nallur, S., et al. (2011). A 3′-untranslated region KRAS variant and triple-negative breast cancer: a case-control and genetic analysis. Lancet Oncol. *12*, 377–386.

53. You, C., Liang, H., Sun, W., Li, J., Liu, Y., Fan, Q., Zhang, H., Yue, X., Li, J., Chen, X., and Ba, Y. (2016). Deregulation of the miR-16-KRAS axis promotes colorectal cancer. Sci. Rep. *6*, 37459.

54. Zhou, Y., Dang, J., Chang, K.Y., Yau, E., Aza-Blanc, P., Moscat, J., and Rana, T.M. (2016). miR-1298 Inhibits Mutant KRAS-Driven Tumor Growth by Repressing FAK and LAMB3. Cancer Res. *76*, 5777–5787.

55. Chen, X., Guo, X., Zhang, H., Xiang, Y., Chen, J., Yin, Y., Cai, X., Wang, K., Wang, G., Ba, Y., et al. (2009). Role of miR-143 targeting KRAS in colorectal tumorigenesis. Oncogene *28*, 1385–1392.

56. Johnson, S.M., Grosshans, H., Shingara, J., Byrom, M., Jarvis, R., Cheng, A., Labourier, E., Reinert, K.L., Brown, D., and Slack, F.J. (2005). RAS is regulated by the let-7 microRNA family. Cell *120*, 635–647.

57. Cancer Genome Atlas Network (2012). Comprehensive molecular portraits of human breast tumours. Nature *490*, 61–70.

58. Ciriello, G., Gatza, M.L., Beck, A.H., Wilkerson, M.D., Rhie, S.K., Pastore, A., Zhang, H., McLellan, M., Yau, C., Kandoth, C., et al.; TCGA Research Network (2015). Comprehensive Molecular Portraits of Invasive Lobular Breast Cancer. Cell *163*, 506–519.

59. Cancer Genome Atlas Research Network (2017). Integrated Genomic Characterization of Pancreatic Ductal Adenocarcinoma. Cancer Cell *32*, 185–203.e13.

60. Zhang, G., Schetter, A., He, P., Funamizu, N., Gaedcke, J., Ghadimi, B.M., Ried, T., Hassan, R., Yfantis, H.G., Lee, D.H., et al. (2012). DPEP1 inhibits tumor cell invasiveness, enhances chemosensitivity and predicts clinical outcome in pancreatic ductal adenocarcinoma. PLoS ONE *7*, e31507.

61. Bayraktar, R., Pichler, M., Kanlikilicer, P., Ivan, C., Bayraktar, E., Kahraman, N., Aslan, B., Oguztuzun, S., Ulasli, M., Arslan, A., et al. (2017). MicroRNA 603 acts as a tumor suppressor and inhibits triple-negative breast cancer tumorigenesis by targeting elongation factor 2 kinase. Oncotarget *8*, 11641–11658.

62. Gonzalez-Villasana, V., Rodriguez-Aguayo, C., Arumugam, T., Cruz-Monserrate, Z., Fuentes-Mattei, E., Deng, D., Hwang, R.F., Wang, H., Ivan, C., Garza, R.J., et al. (2014). Bisphosphonates inhibit stellate cell activity and enhance antitumor effects of nanoparticle albumin-bound paclitaxel in pancreatic ductal adenocarcinoma. Mol. Cancer Ther. *13*, 2583–2594.

63. Bayraktar, R., Ivan, C., Bayraktar, E., Kanlikilicer, P., Kabil, N.N., Kahraman, N., Mokhlis, H.A., Karakas, D., Rodriguez-Aguayo, C., Arslan, A., et al. (2018). Dual suppressive effect of miR-34a on the FOXM1/eEF2-kinase axis regulates triple-negative breast cancer growth and invasion. Clin. Cancer Res. *24*, 4225–4241.

64. Hamurcu, Z., Kahraman, N., Ashour, A., and Ozpolat, B. (2017). FOXM1 transcriptionally regulates expression of integrin β1 in triple-negative breast cancer. Breast Cancer Res. Treat. *163*, 485–493.

OMTN, Volume *14*

Supplemental Information

The Modulatory Role of MicroRNA-873 in the Progression of KRAS-Driven Cancers

Hamada A. Mokhlis, Recep Bayraktar, Nashwa N. Kabil, Ayse Caner, Nermin Kahraman, Cristian Rodriguez-Aguayo, Erika P. Zambalde, Jianting Sheng, Kübra Karagoz, Pinar Kanlikilicer, Abdel Aziz H. Abdel Aziz, Tamer M. Abdelghany, Ahmed A. Ashour, Stephen Wong, Michael L. Gatza, George A. Calin, Gabriel Lopez-Berestein, and Bulent Ozpolat

Supplementary Data

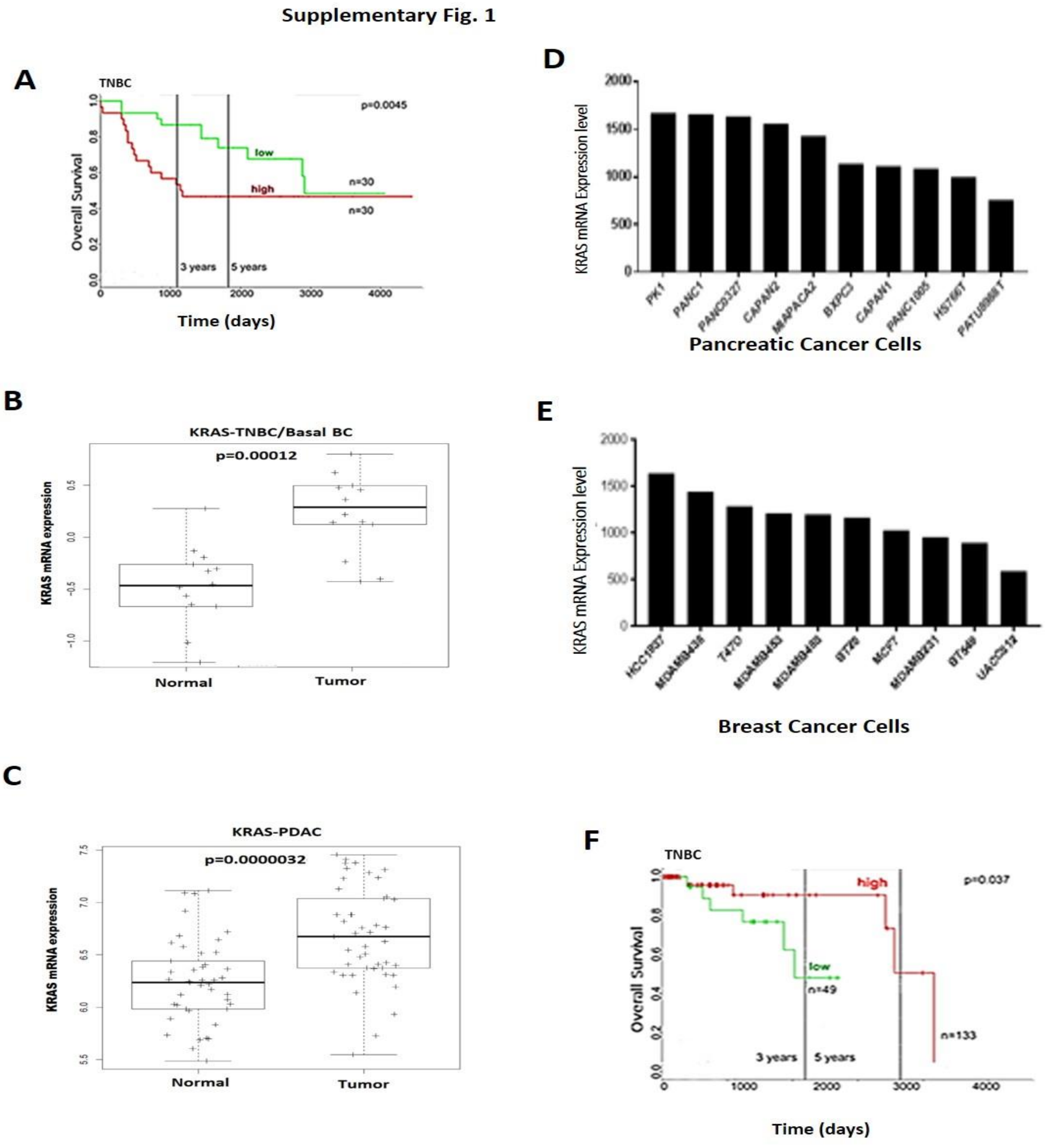

Supplementary Figure 1: KRAS is frequently upregulated in pancreatic and Breast cancer tissues. (A) Low KRAS mRNA expression is associated with high overall survival in patients with TNBC (n = 60, p = 0.0045). Data of TNBC were obtained from the PROGgeneV2 database. **(B)** The expression level of

KRAS mRNA in 45 pancreatic cancer tissues versus 45 normal tissues according to the GEO database (GSE28735), P=0.0000032. **(C)** The expression level of KRAS mRNA in 14 basal-like breast cancer tissues versus 14 normal breast tissues according to TCGA database, p=0.00012. **(D, E)** Analysis of Cancer Cell Line Encyclopedia (CCLE) database for KRAS expression profile in PDAC and BC cells. CCLE converted raw Affymetrix CEL files to a single value for each probeset using the robust multi-array average (RMA) and quantile normalization. PANC1, MiaPaCa-2, MDA-MB-231, and MDA-MB-436 cells are selected for further experiments based on the cell expression data. **(F)** High miR-873 expression is associated with high overall survival in patients with TNBC (n= 182, p = 0.037). Data of TNBC were obtained from miRpower tool (http://kmplot.com/analysis/).

Supplementary Fig. 2

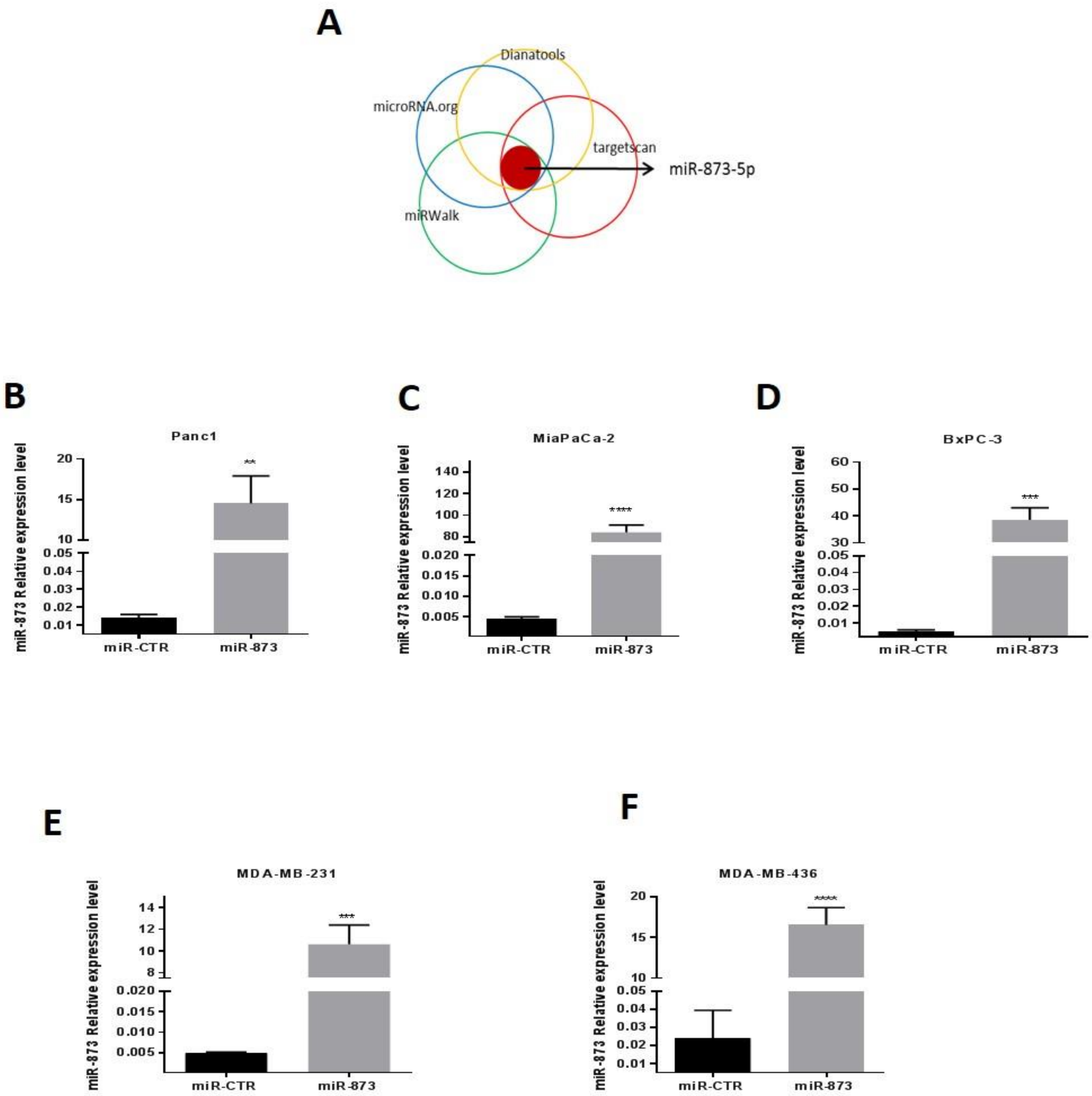

Supplementary Figure 2. Ectopic expression of miR-873 increases relative miR-873 expression levels in pancreatic ductal adenocarcinoma (PDAC) and triple-negative breast cancer (TNBC) cells. (A) Computational algorithms, including Targetscan, Diana tools microRNA.org and miRWalk2.0 that predict the potential of microRNAs binding to the 3′

untranslated region of KRAS mRNA. Among the potential target mRNAs, miR-873-5p was identified. **(B-F)** Cells were transfected with miR-873-100nM or control mimic for 72 h and miR-873 expression levels were analyzed in PDAC and TNBC cells using quantitative reverse-transcriptase polymerase chain reaction. Data represent means + standard error of three independent experiments. **p < 0.01; ***p < 0.001; ****p < 0.0001.

Supplementary Fig. 3

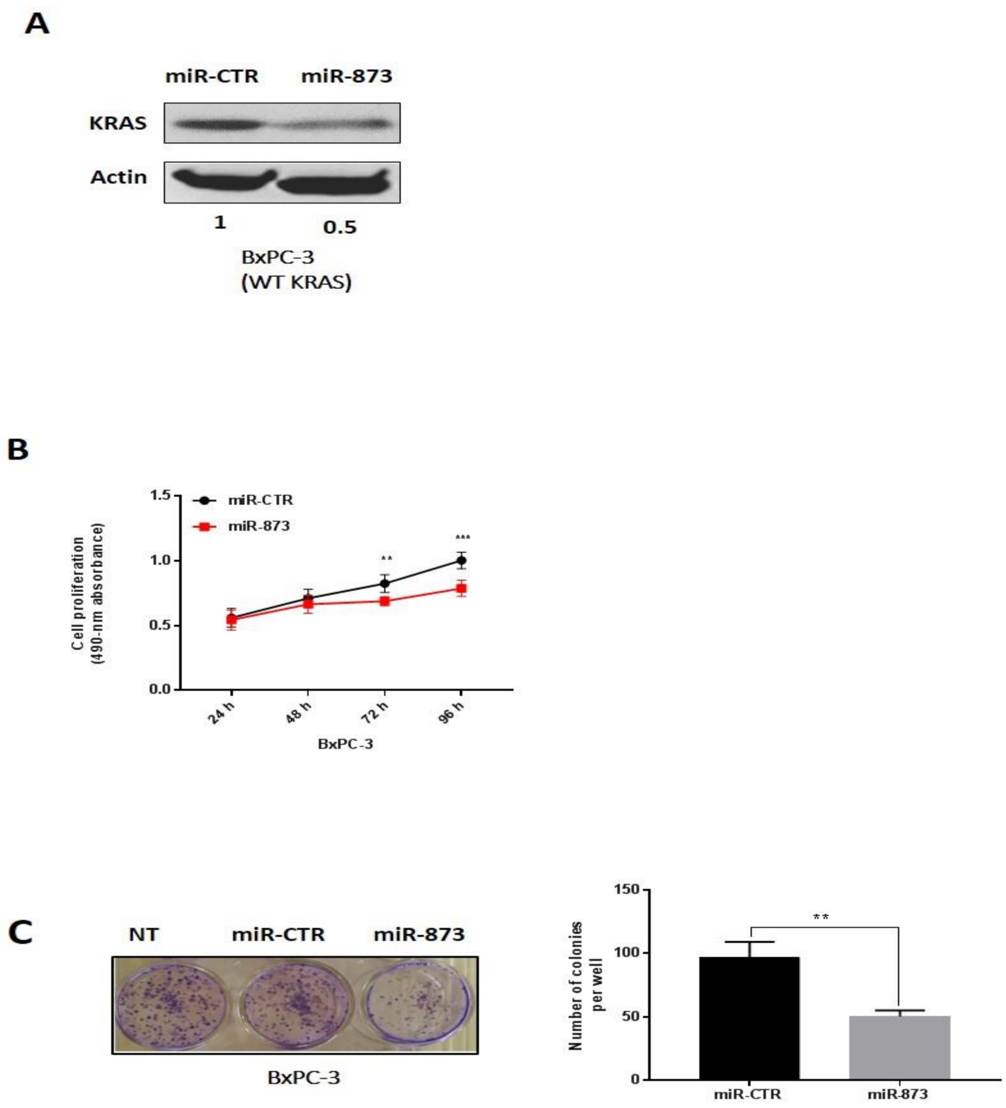

Supplementary Figure 3. Ectopic expression of miR-873 inhibits KRAS in pancreatic ductal adenocarcinoma cells harboring wild-type (WT) KRAS. (A) BxPC-3 cells were transfected with miR-873 or control mimic for 48 h and KRAS expression levels were analyzed by Western blot. (**B, C**) miR-873 suppresses cell proliferation (**B**) and colony formation (**C**) in BxPC-3 cells. Data represent means + standard error of three independent experiments. $^{**}p < 0.01$; $^{***}p < 0.001$ (compared with control group).

Supplementary Fig. 4

A

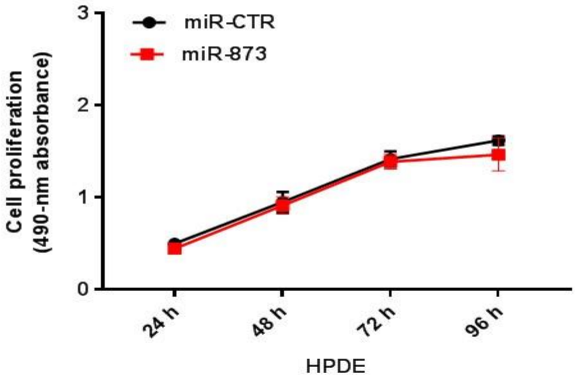

B

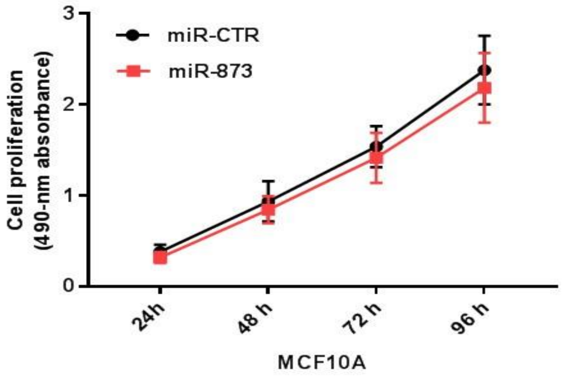

Supplementary Figure 4. MiR-873 has no remarkable effect on the proliferation of normal cells. The short-term effects of ectopic expression of miR-873 on the viability of normal pancreatic HPDE cells (**A**) and normal breast epithelial MCF10A cells (**B**) were examined using the MTS assay. The mean absorbance at 490 nm was determined at 48, 72, and 96 h. Data represent means + standard error of three independent experiments.

Supplementary Fig. 5

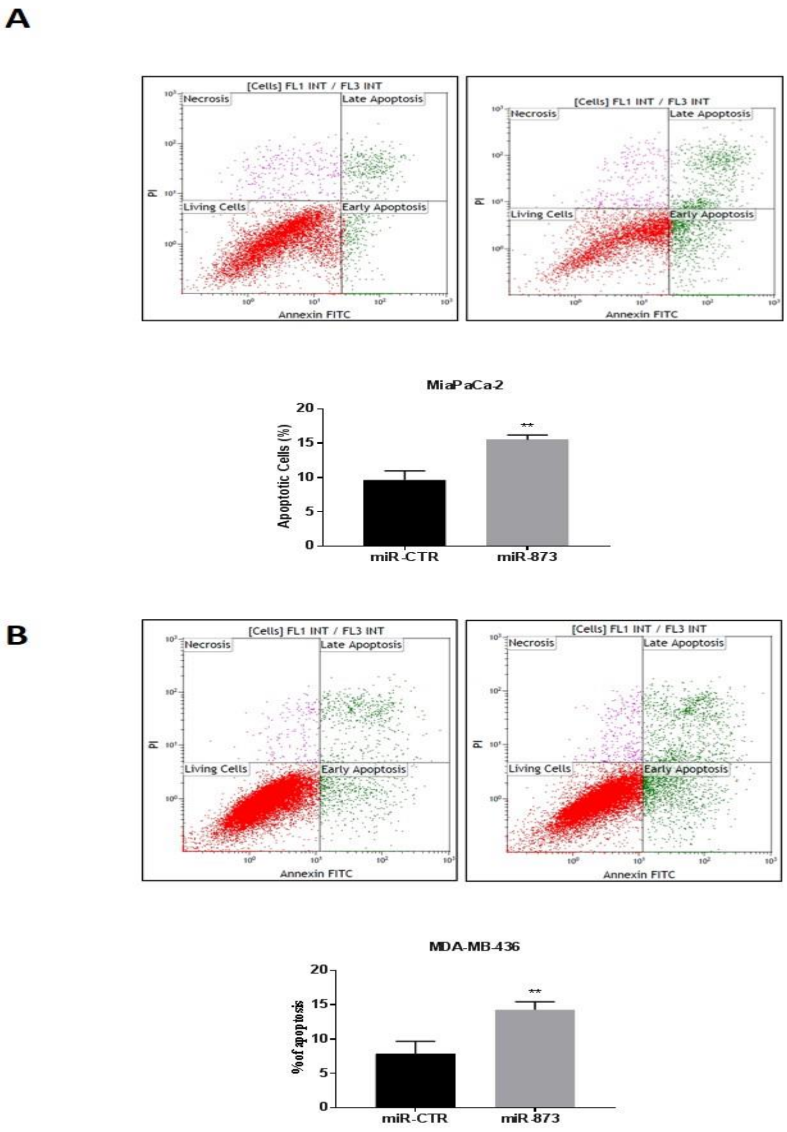

Supplementary Figure 5. MiR-873 triggers apoptosis in MiaPaCa-2 and MDA-MB-436 cells. miR-873 mimic or control mimic were transfected in MiaPaCa-2 **(A)** and MDA-MB-436 **(B)**, after 72h, cells were analyzed by Annexin V-FITC and PI double-staining and positive cells were detected and quantified by FACS analysis. The represented percentages show positive cells at both early and late apoptosis. Data are represented as mean ± SE. ** $P < 0.01$ indicates a significant difference compared with control group.

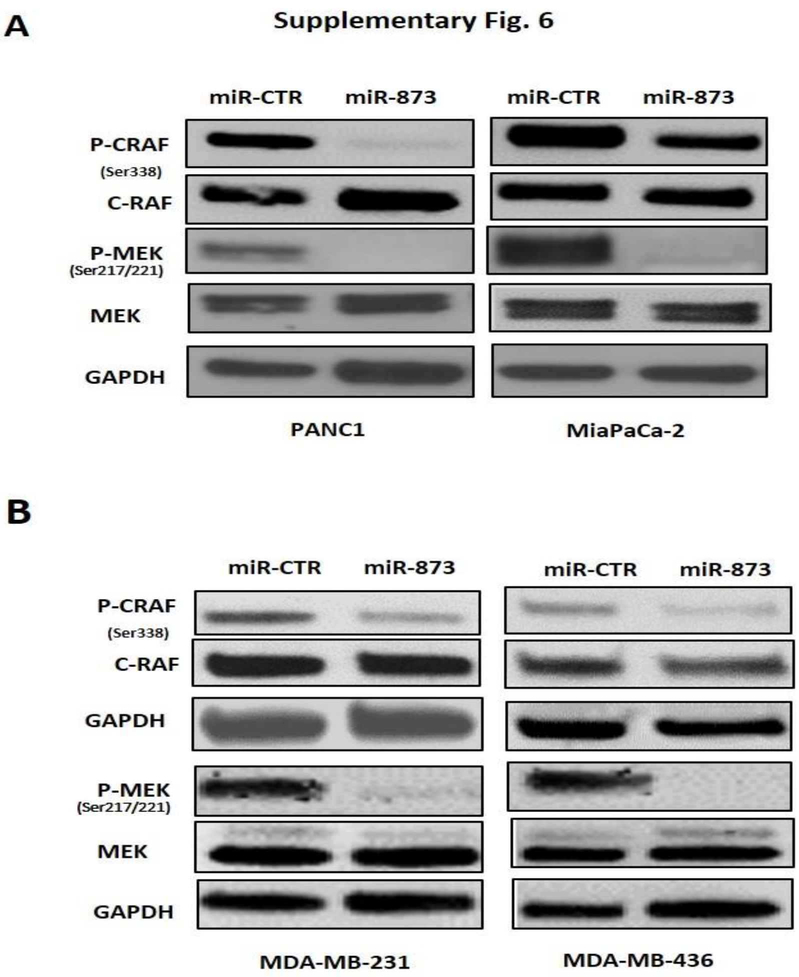

Supplementary Figure 6. MiR-873 inhibits KRAS downstream effectors.

Overexpression of miR-873 inhibited p-C-RAF and p-MEK1/2 in both PANC1, MiaPaCa-2 cells **(A)**, MDA-MB-231 and MDA-MB-436 **(B)**.

Supplementary Fig. 7

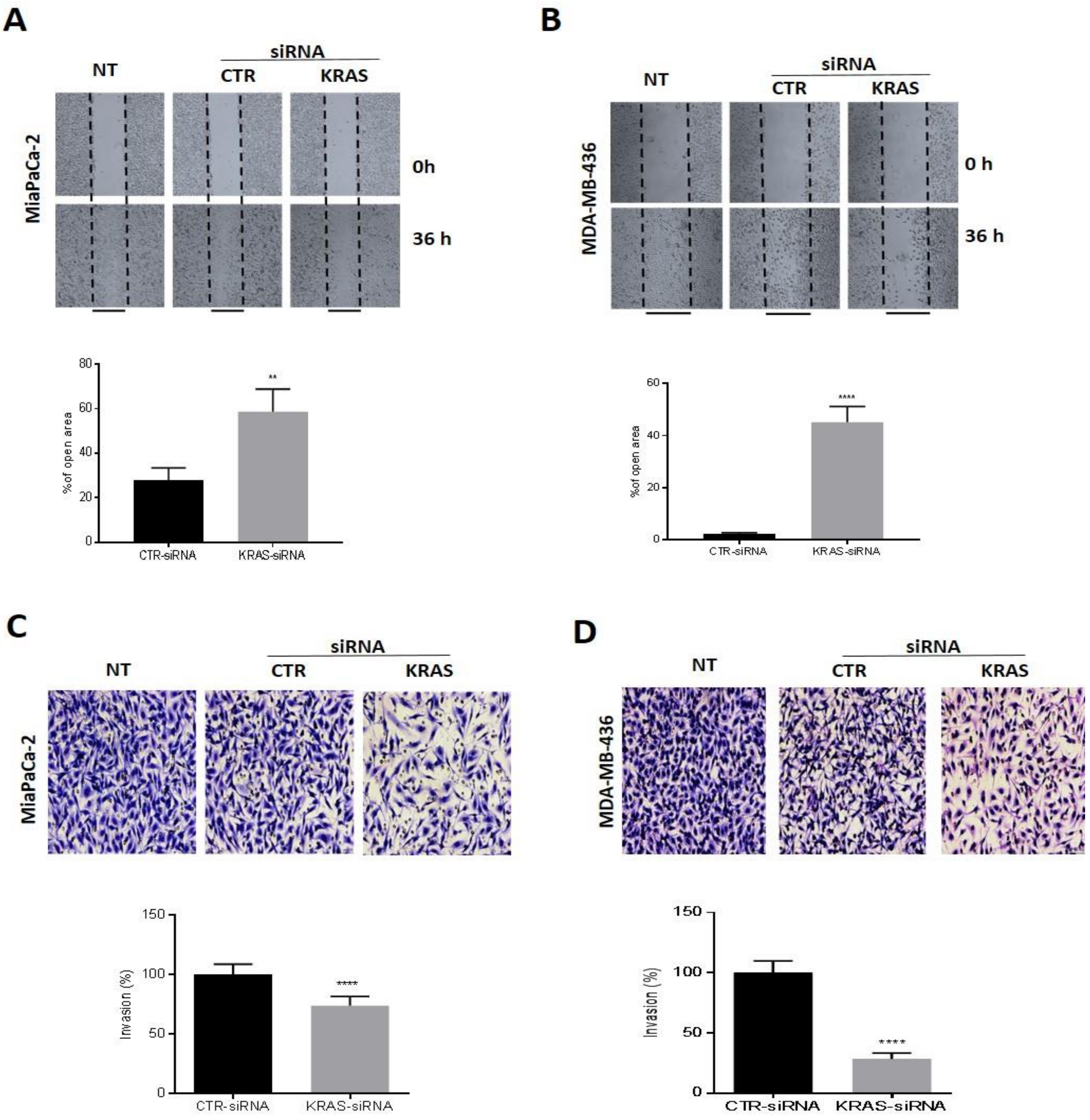

Supplementary Figure 7. Knockdown of KRAS by siRNA leads to inhibition of cell migration and invasion in vitro. Silencing of KRAS in PDAC (MiaPaCa-2) and TNBC (MDA-MB-436) cells led to a significant inhibition of cell migration **(A, B)** and invasion **(C, D)**. Data represent means + standard error of three independent experiments. $^{**}p < 0.01$; $^{****}p < 0.0001$.

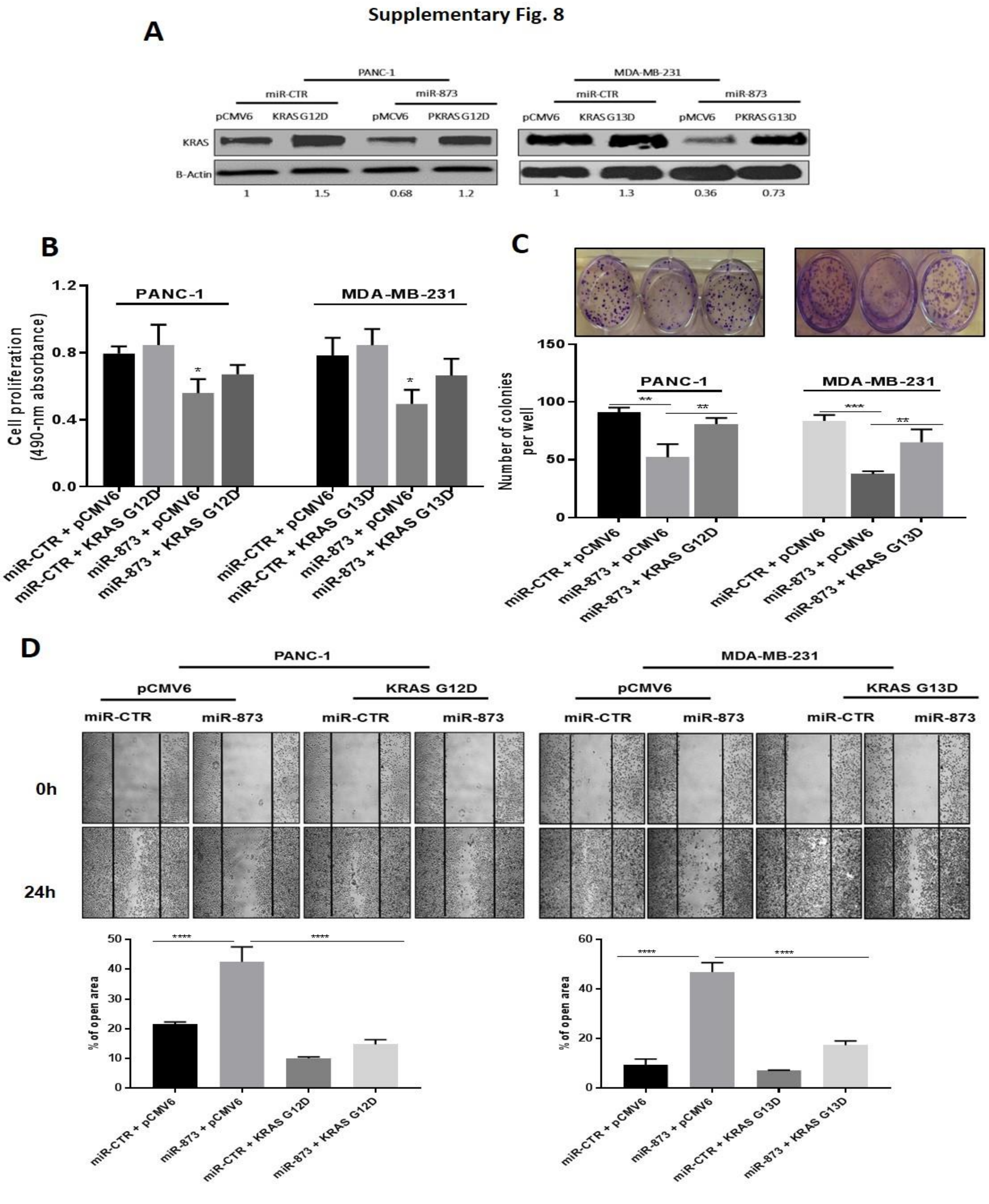

Supplementary Figure 8. Overexpression of KRAS rescues the effects of miR-873 on PDAC and TNBC cells. (A) Cells were transfected with the KRAS mutated clone vector -G12D for

PANC-1 and G13D for MDA-MB-231- and /or pMCV6 empty vector, then cells were transfected with miR-873 or control mimic and were analyzed by Western blot 48 h later for the expression of KRAS. β-actin was used as a loading control. (**B, C)** Overexpression of KRAS in PANC-1 and MDA-MB-231 cells reversed the effects of miR-873 mimic treatment on cell proliferation and clonogenic cell growth. **(D)** KRAS overexpression reversed the effects of miR-873-induced inhibition of cell migration in PANC-1 and MDA-MB-231 cells. Data represent means + standard error of three independent experiments. * $P < 0.05$ **$p < 0.01$; *** $P < 0.001$ ****$p < 0.0001$.

Supplementary Fig. 9

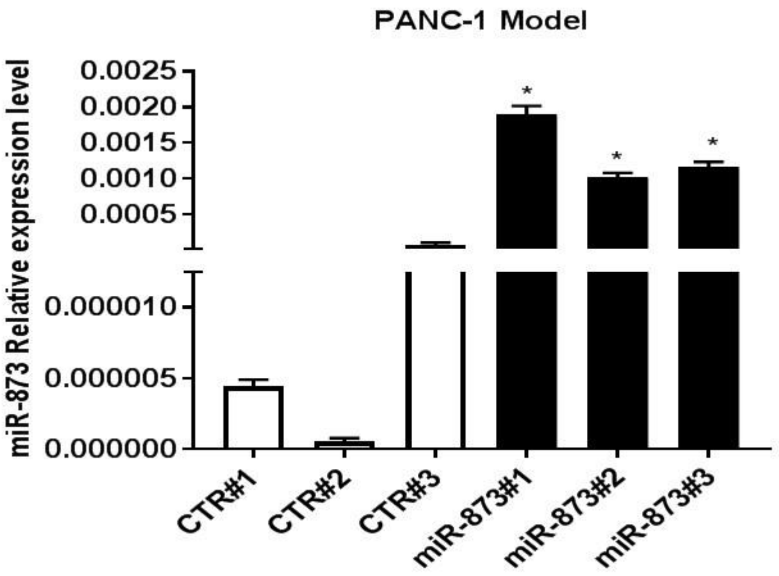

Supplementary Figure 9. Effects of in vivo treatment with nanoliposomal miR-873 on the weight of mice bearing pancreatic ductal adenocarcinoma (PDAC) and triple-negative breast cancer (TNBC) tumors. In mice with PDAC PANC1 **(A)** and TNBC MDA-MB-231 **(B)** xenografts, treatment with miR-873 for 4 to 5 weeks did not cause any change in the mean mouse weight after 4 weeks. Data represent means + standard error.

Supplementary Fig. 10

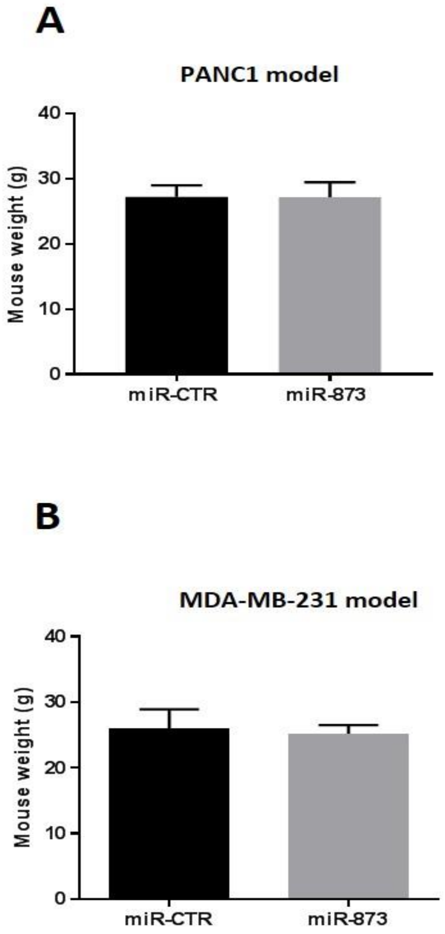

Supplementary Figure 10. Confirmation of successful delivery of miR-873 into tumor tissues. PANC-1 cells were injected into female nude mice (n=5). After tumor notice, mice were then treated with either miR-CTR or miR-873 mimic liposomal nano-particles intravenously once every 4 days, for 5 weeks. Tumor tissues were processed and total RNA was isolated from three random samples per each group and level of miR-873 was determined in those samples. U6 was utilized as an internal control. Mice treated with miR-873 showed higher expression levels of miR-873 than those treated with miR-CTR. Data represent means + standard error. * $P < 0.05$

Supplementary Fig. 11

A

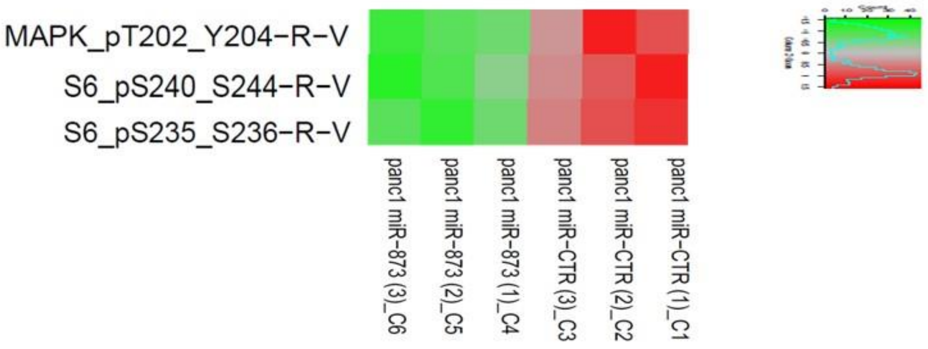

B

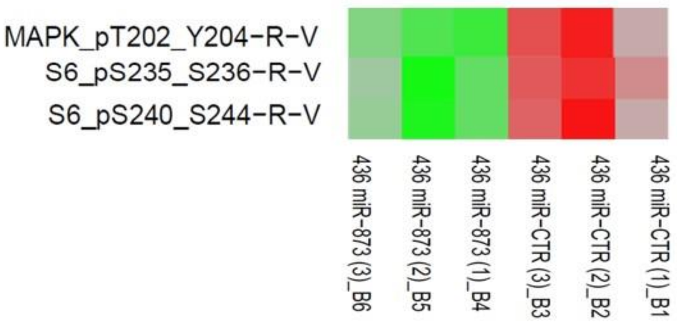

Supplementary Figure 11. Reverse phase protein array (RPPA) results reveal an array of altered proteins in PANC1 and MDA-MB-436 cells. (A, B) The heat map of RPPA analysis shows the results, with significant changes in cells after treatment with miR-873 and control miRNA. The expression ratios for a given sample group of interest were represented by their mean. Rows, proteins; columns, signal ratios of miR-873– or control miRNA–transfected PANC1 and MDA-MB-436 cells. For each protein, red indicates that the expression level of that protein was higher in miR-873–transfected cells than in control miRNA–transfected cells, and green indicates that the expression level was lower.

Supplementary Table 1: Median survival of pancreatic cancer patients and basal-like breast cancer patients.

Supplementary Table 1:

Pancreatic cancer		Median Survival
	Low KRAS	23.4
	High KRAS	18.7
	Low mir-873	19.7
	High mir-8A73	22.8
Basal like breast cancer		
	Low KRAS	115.7
	High KRAS	Undefined (>180)
	Low mir-873	Undefined (>55)
	High mir-873	115.7
